# Large-scale transcriptome data reveals transcriptional activity of fission yeast LTR retrotransposons

**DOI:** 10.1186/1471-2164-11-167

**Published:** 2010-03-12

**Authors:** Tobias Mourier, Eske Willerslev

**Affiliations:** 1 Ancient DNA and Evolution Group, Natural History Museum of Denmark, University of Copenhagen, Øster Voldgade 5-7, DK-1350 Copenhagen, Denmark

## Abstract

**Background:**

Retrotransposons are transposable elements that proliferate within eukaryotic genomes through a process involving reverse transcription. The numbers of retrotransposons within genomes and differences between closely related species may yield insight into the evolutionary history of the elements. Less is known about the ongoing dynamics of retrotransposons, as analysis of genome sequences will only reveal insertions of retrotransposons that are fixed - or near fixation - in the population or strain from which genetic material has been extracted for sequencing. One pre-requisite for retrotransposition is transcription of the elements. Given their intrinsic sequence redundancy, transcriptome-level analyses of transposable elements are scarce. We have used recently published transcriptome data from the fission yeast *Schizosaccharomyces pombe *to assess the ability to detect and describe transcriptional activity from Long Terminal Repeat (LTR) retrotransposons. LTR retrotransposons are normally flanked by two LTR sequences. However, the majority of LTR sequences in *S. pombe *exist as solitary LTRs, i.e. as single terminal repeat sequences not flanking a retrotransposon. Transcriptional activity was analysed for both full-length LTR retrotransposons and solitary LTRs.

**Results:**

Two independent sets of transcriptome data reveal the presence of full-length, polyadenylated transcripts from LTR retrotransposons in *S. pombe *during growth phase in rich medium. The redundancy of retrotransposon sequences makes it difficult to assess which elements are transcriptionally active, but data strongly indicates that only a subset of the LTR retrotransposons contribute significantly to the detected transcription. A considerable level of reverse strand transcription is also detected. Equal levels of transcriptional activity are observed from both strands of solitary LTR sequences. Transcriptome data collected during meiosis suggests that transcription of solitary LTRs is correlated with the transcription of nearby protein-coding genes.

**Conclusions:**

Presumably, the host organism negatively regulates proliferation of LTR retrotransposons. The finding of considerable transcriptional activity of retrotransposons suggests that part of this regulation is likely to take place at a post-transcriptional level. Alternatively, the transcriptional activity may signify a hitherto unrecognized activity level of retrotransposon proliferation. Our findings underline the usefulness of transcriptome data in elucidating dynamics in retrotransposon transcription.

## Background

With only a few exceptions [1,2], retrotransposons have been found in all analysed eukaryotic genomes. Although transcription of retrotransposons is an integral part of their life cycle, elements may be transcriptionally active without this resulting in proliferation of the elements within the host genome [3,4]. Transcriptional activity of retrotransposons has been detected in a range of organisms and conditions, and may involve a multitude of elements that are simultaneously transcribed [5-7], or alternatively, single element loci driving transcription of nearby genes [8,9]. The presence and transcriptional activity of retrotransposons may interfere with nearby genes [10], and hence presumably are subject to negative selection [11-13].

Unfortunately, the intrinsic sequence redundancy of retrotransposons has limited the resolution by which activity can be assigned to specific elements (or classes thereof) using genome-scale approaches [14,15]. The recent advances in novel sequencing and hybridization technologies [16,17] have permitted an unforeseen depth in detection of transcriptional activity. Recently, Faulkner and colleagues reported that 6-30% of cap-selected mammalian transcripts were initiated in repetitive elements [4]. We set out to test if transcriptome data could provide information on the transcriptional activity of presumably functional (i.e. retrotransposition-competent) retrotransposons, and turned our attention to the single-celled fission yeast *Schizosaccharomyces pombe*. The genome of *S. pombe *is highly compact and well annotated [18], and harbours only a few families of Long Terminal Repeat (LTR) retrotransposons [19,20]. LTR retrotransposons are transposable elements that typically contain *gag *and *pol *genes required for transposition, are related to retroviruses, and have their name from the two repeated LTR sequences flanking them. Two LTR sequences may recombine resulting in a solitary LTR sequence. All full-length LTR retrotransposons in the *S. pombe *reference genome (strain 972) belong to the Tf2 family, while all members of the other dominant LTR family, Tf1, are found as solitary LTR sequences [18,21]. *S. pombe *LTR elements are predominantly inserted upstream of protein-coding genes [22,23], where transcription activators are responsible for targeting the site of insertion [24]. Intriguingly, the Tf1 elements were shown to harbour promoter regions restoring the regulatory functions that are disrupted by LTR integration [24].

We have analysed the data from two recent studies: First, a high throughput sequencing of complementary DNAs generating short reads (30-51 nucleotides) from *S. pombe *growth phase and five time points during meiosis from the Bähler lab [25]. This study is henceforth referred to as RNA-Seq. Second, a study from the Cairns lab [26] using a novel approach (called HybMap) in which RNA from growth phase was directly hybridized to a whole-genome microarray with 60 base pair DNA probes, followed by antibody procedures ensuring perfect matches as well as subsequent quantification by light emission [16]. The direct hybridization approach hence allows the assignment of transcriptional activity to a specific strand. We refer to this study simply as HybMap. The available data samples are summarized in Additional file 1; Table S1. From RNA-Seq sequence reads and signal intensities of HybMap array probes we recorded the transcriptional activity from LTR sequences, analysed the extent and orientation of LTR transcription, and how the expression profiles of LTR sequences correlate with nearby genes.

## Results and discussion

We retrieved 239 LTR sequences with lengths from 75 to 412 base pairs (bp) (median/average size: 346/321 bp) from the *S. pombe *genome annotation. Of these, 25 LTR sequences were residing in full-length LTR retrotransposons. This uneven number results from a single chimerical LTR retrotransposon with an [LTR-internal sequence-LTR-internal sequence-LTR] organization that potentially is a result of an ectopic recombination event. Two full-length LTR retrotransposons (SPBC1E8.04 and SPCC1494.11c) are frame shifted and annotated as pseudogenes http://www.genedb.org/. Genomic coordinates of the LTR sequences are provided in Additional file 1; Table S2. From a biological point of view, we are interested in distinguishing between transcriptional activity stemming from full-length LTR retrotransposons and from solitary LTRs. Analysis of transcriptional activity was therefore performed on these two sets of LTRs separately: 13 full-length LTR retrotransposons (each consisting of the internal sequence flanked by LTR sequences) and the remaining 214 solitary LTR sequences.

**Figure 1:**
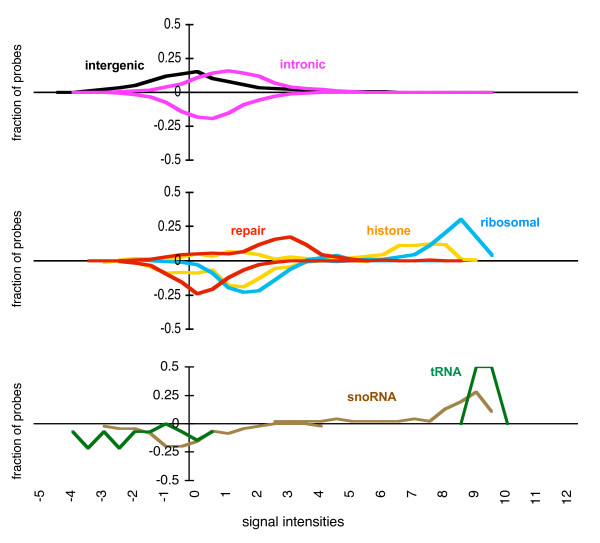
**HybMap signal intensities for genomic features**. The distributions of signal intensities for HybMap probes mapping to different genomic features - as indicated on the figure - are shown. Probes were grouped into bins according to their signal intensities (as shown on the x-axis) and the fraction of probes belonging to each bin is shown on the y-axis. For clarity, distributions are split into three separate charts. Signal intensities are log2 normalized against a background intensity [26]. Intensities for probes mapping to the forward strand are shown above x-axes, and to the reverse strand below x-axes. The absolute numbers of probes are provided in Table 1. Signals values are shown below the bottom panel only, but are identical for all sections.

To assess the level of transcriptional activity using the HybMap approach, we collected the recorded signal intensities of HybMap probes mapping exclusively within the full-length LTR set and uniquely within the solitary LTR sets, and compared these to signal intensities for probes mapping to other genomic reference features. These reference features include RNA genes, protein-coding genes and background sequences (intergenic and intronic) (Table 1). As expected, when plotting the signal intensity for genes (both protein-coding and RNA) we observe higher levels of signal intensities for forward strand probes, and lower levels of intensities for reverse strand probes (Figure 1). Signal intensities for intergenic mapping probes are distributed around zero, in accordance with the normalization procedures carried out in the HybMap study [26]. Finally, low levels of signals are observed for intronic mapping probes, showing slightly higher intensities on the forward than on the reverse strands. Signal intensities for the two LTR sets are presented in detail below.

**Table 1:** Data sets of LTR sequence sets and other genomic features

Genomic features	Seqs^a^	Total size (bp)	HybMap probes^b^	RNA-Seq reads^c^
LTRs				

Full-length LTR elements	13	63855	1032^d^	901^d^
Solitary LTRs	214	67938	1298	817^d^

Background				

Intergenic	3206	2796112	59528	300955
Intronic	943	177104	4380	6939

RNA genes				

tRNAs	170	13130	28	290
snoRNAs	23	4073	91	2941

Highly expressed protein-coding genes				

Histone proteins	12	12964	422	8672
Ribosomal proteins	124	62661	1794	236760

Lowly expressed protein-coding genes				

Repair genes (rec and rad)	43	82258	2860	10833

a) Number of sequences in set

b) HybMap 60-mers mapping to sequence sets. Both forward and reverse strand probes included.

c) RNA-Seq sequence reads from growth stage mapping to sequence sets.

d) Number provided is probes and reads mapping exclusively within sequence sets (otherwise these are mapping uniquely)

### Full-length LTR retrotransposons

**Figure 2:**
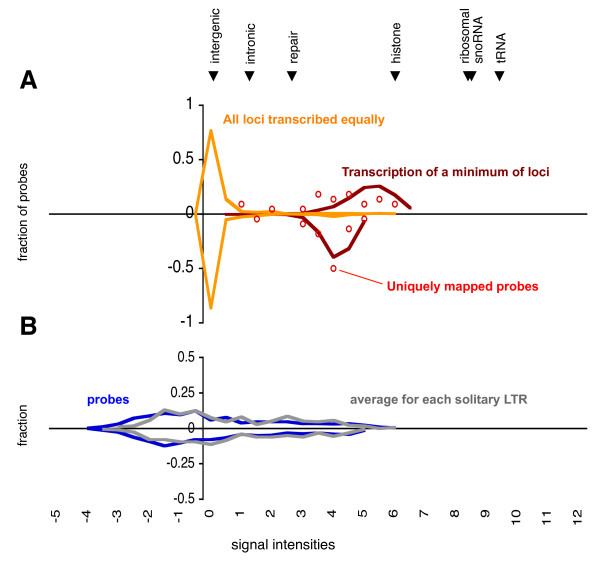
**HybMap signal intensities for LTR sequences**. The distributions of signal intensities for HybMap probes mapping to full-length LTR retrotransposons (A) and solitary LTRs (B). General figure format as in Figure 1. **A**) Intensity distribution of full-length LTR probes are displayed using two different procedures: i) each probe's intensity divided by the number of possible mappings (assuming all LTR loci being transcribed equally; yellow curve), ii) total intensity of each probe assigned to one locus (transcription of a minimum of loci; red curve). The intensity distribution of probes mapping uniquely to a single full-length LTR retrotransposon locus are shown as open red circles. **B**) Intensity distribution for probes mapping uniquely to solitary LTR sequences (blue curve). For each solitary LTR loci the average intensity was calculated and plotted (grey curve). For comparison, the median intensity of forward probes mapping to other genomic features are indicated at the top of the figure.

A total of 1032 (516 on each strand) HybMap probes are mapping exclusively to the set of full-length LTR retrotransposons. Of these, only 44 probes (22 on each strand) are mapping uniquely to a single retrotransposon locus (Additional file 2; Figure S1). This means that for the vast majority of probes we cannot determine the number of loci contributing to any transcriptional activity. In Figure 2A, yellow curves depict the scenario where all retrotransposons contribute equally (i.e. each probe intensity is divided by its number of possible mappings), and red curves depict the other extreme where the total signal of each probe is derived from a single loci. As seen from Figure 2A, the former scenario indicates that LTR retrotransposons are not transcribed at a level exceeding that of background transcription of intergenic sequence, whereas the latter scenario is equivalent to a few active LTR retrotransposon transcribed at a level comparable to histone genes. When plotting the signal intensities of uniquely mapping probes (red circles in Figure 2), we see that these are highly skewed towards higher intensities, suggesting that only a small number of LTR retrotransposon loci contribute to the combined transcriptional activity, and concurrently, that these retrotransposons are transcribed at high levels. The full-length uniquely mapping probes target 5 different LTR retrotransposons, of which 2 are the aforementioned pseudogenes (Additional file 2; Figure S1). Thus, transcription is detectable from at least 3 retrotransposons with complete open reading frames. We cannot, however, conclude anything about the individual levels of transcriptional activity from the remaining 8 full-length LTR retrotransposons that do not have any unique probes assigned to them.

**Figure 3:**
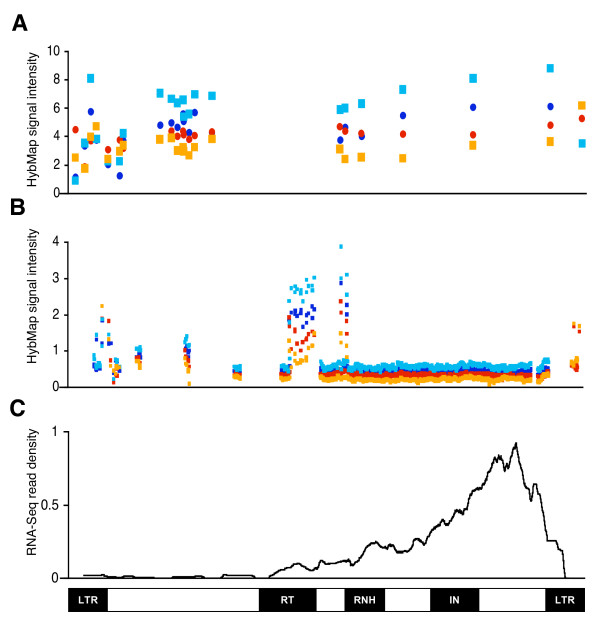
**Transcriptional activity along full-length LTR retrotransposons**. **A**) Signal intensities of HybMap probes (y-axis) mapping uniquely to a single full-length LTR locus are plotted along the retrotransposon sequence (x-axis). Total RNA samples are shown as blue circles (forward strand probes) and red circles (reverse strand probes). Poly(A)-enriched samples are shown as light blue squares (forward) and orange squares (reverse). **B**) As above, but with probes mapping to multiple full-length LTR retrotransposons. **C**) The density of RNA-Seq reads mapping to the LTRs are shown as the average using a 301-bp long sliding window. Density shown as reads mapped per sequence in the alignment. The genetic structure of an LTR retrotransposon is depicted at the bottom, with flanking LTR sequences, as well as reverse transcriptase (RT), RNaseH (RNH) and integrase (IN) domains indicated.

We next mapped HybMap probes onto an alignment of the full-length LTR retrotransposons (Figure 3A&3B). Although data shown in Figure 2 strongly suggest that a minority of LTR retrotransposons contribute to the overall transcriptional activity, in Figure 3 probe intensities are divided by the number of possible mappings (i.e. following the assumption that all LTR loci are equally active). When ignoring the differences between those probes that are mapping uniquely and those that are not, transcription appears to be relatively equal across the retrotransposon internal sequence, consistent with transcription of the entire retrotransposon. The notable exceptions of two areas covering parts of the Reverse Transcriptase and RNase H domains (Figure 3A&3B) coincide with regions where probes are mapping to a lower number of full-length LTR retrotransposons (Additional file 2; Figure S1), providing additional support for the notion of a few highly transcriptionally active retrotransposons. Although high levels of reverse strand transcription is apparent, poly(A)-enriched samples display a better resolution between the two strands, suggesting that polyadenylated full-length LTR retrotransposon transcripts are present in the samples (Figure 3A&3B).

The fact that two almost identical flanking LTRs are present in each full-length retrotransposon, and the likelihood that an LTR mapping probe will also map to LTR sequences outside the set of full-length elements (and hence be excluded by our procedure), makes it difficult to compare directly the flanking LTRs and the internal sequence. We would expect transcription to initiate at the transcription start site in the 5' LTR (roughly halfway into the LTR sequence [20]) and terminate in the 3' LTR. In reality, as LTR-matching probes are often mapping to both flanking LTR sequences, we cannot establish from which end the activity stems, and for consistency we have simply mapped such probes to the 3' LTR in Figure 3. Interestingly, no clear distinction between forward and reverse strand transcriptional activity is observed for the LTR sequences.

### Solitary LTR sequences

Transcriptional activity is also detectable from solitary LTR sequences, although only a subset of the 1298 probes mapping uniquely to solitary LTRs shows high levels of signal intensities (Figure 2B, blue curve). Further, transcriptional activity is similar from the forward and the reverse strand. To establish if the divergent intensities of signals from solitary LTR probes are a result of differences between LTR loci, or between probes within LTR sequences, we calculated the average signal intensity for each LTR locus. If the observed broad range of signal intensities in Figure 2B was a result of LTR loci with equal levels of transcriptional activity, but with certain parts of the LTR sequences being transcribed and some not, we would expect the average signal intensity for individual LTR sequences to be relatively similar. This is not the case (Figure 2B, grey curve), suggesting that individual solitary LTR loci differ in levels of transcriptional activity. Then why are some LTR loci apparently transcribed whereas others are not? We do not find any noticeable patterns between highly and lowly transcribed LTR sequences in terms of orientation and distance with respect to nearest neighbouring protein-coding gene (not shown). In fact, no correlation is found between the average signal intensity of LTR sequences and the average of the nearest gene (Additional file 2; Figure S2). Finally, to test if the observed transcriptional differences between specific LTR sequences are due to simple stochastic variation due to the relatively small sample size, we performed a permutation analysis in which probe intensities were randomly assigned to an LTR sequence (while keeping the distribution of uniquely mapping probes to LTRs constant, see Methods). When repeating this analysis 10.000 times, we find that the observed variance between the average signal intensities from each LTR sequence (forward variance: 2.08, reverse variance 2.03) fall well without the range of variances of the simulated sets (forward range 1.08-1.67, reverse range 1.11-1.65) (Additional file 2; Figure S3). This strongly suggests that distinct differences in transcriptional activity levels exist between *S. pombe *solitary LTR sequence loci.

**Figure 4:**
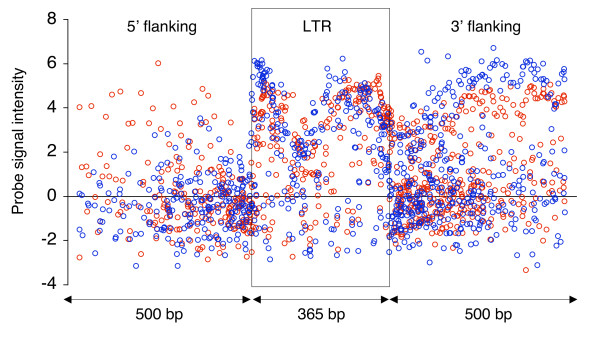
**Transcriptional profiles of solitary LTR sequences**. Transcriptional activity across 71 solitary LTR sequences and a maximum of 500 bp upstream and downstream (5' flanking and 3' flanking, respectively). The borders of the LTR sequences are indicated by the black box. HybMap probes are plotted according to their mapped position and their intensity signal (y-axis). Forward strand probes shown as blue circles, reverse strand probes as red circles.

Transcriptional activity stemming from solitary LTRs could be a result of transcription initiated outside the LTR sequences. To assess this, we included genomic sequence flanking the LTR sequences and compared transcriptional activity within LTR sequences to activity in their genomic vicinity. In practice, we collected a set of 71 easily alignable solitary LTR sequences (see Methods), and for each LTR sequence we collected a maximum of 500 base pair flanking sequence both up- and downstream. If the distance to another annotated feature (e.g. a protein-coding gene) was lower than 1000 bp, only half of this distance was used as flanking sequence. If half the distance to nearest feature was smaller than 60 base pairs no flanking sequence was included. The flanking sequences were then concatenated with the alignment of solitary LTRs, so that flanking sequence was not aligned but fixed by the borders of LTR sequences. A schematic depiction of the procedure and statistics on sizes of flanking sequences are shown in Additional file 2; Figure S4. As seen from Figure 4, a decrease in the level in transcriptional activity is observed immediately outside the LTR sequences. The median level of signal intensities within LTR sequences (median: 2.85) was found to be significantly higher than both flanking sequences upstream (median: -0.38; Mann-Whitney, U = 44186, p < 8.2 × 10^-6^) and downstream (median: 0.39; Mann-Whitney, U = 128404, p < 9.1 × 10^-6^). Therefore, the LTR transcriptional activity as indicated by HybMap probes does not appear to be a result of transcription continuing into LTRs from the flanking genomic regions, but seems remarkably confined to the LTR sequences.

### HybMap analysis from alternative procedures and growth conditions

Data from the Cairns group HybMap study include hybridizations using poly(A)-enriched RNA samples, as well as samples from alternative growth conditions (minimal medium, heat shock and DNA damage) (Additional file 1; Table S1) [26]. In general, alternative growth conditions yield similar patterns of LTR transcriptional activity (Additional file 2; Figure S5). Notably, a relative higher forward strand activity from full-length LTR retrotransposons is found in poly(A)-enriched samples, consistent with polyadenylation of the full-length LTR retrotransposons (Additional file 2; Figure S5). In contrast, signal intensities from solitary LTR sequences are shifted markedly towards lower values in poly(A)-enriched samples, suggesting that no polyadenylation takes place (Additional file 2; Figure S5).

### RNA-Seq analysis of LTR transcriptional activity

Sequence reads from the RNA-Seq study were mapped to the LTR sequences and other genomic features in a manner similar to the HybMap probes. Contrary to the HybMap approach, RNA-Seq involves conversion of sampled RNA into cDNA (in this case using a poly(dT) primer [25]). Presumably this step is responsible for the observed bias of higher RNA-Seq read densities towards the 3' end of the protein-coding genes in our reference set - a bias that is not observed for the HybMap intensities (Additional file 2; Figure S6). Consistent with this, when we plot the RNA-Seq reads mapping exclusively to full-length LTR retrotransposons onto the alignment, a pronounced 3' peak is observed (Figure 3C). Again, this strongly suggests that *S. pombe *LTR retrotransposons are actively transcribed in full length and polyadenylated.

For the full-length LTR elements, we assigned the RNA-Seq reads using two complementary procedures. First, reads mapping to multiple loci were assigned evenly between LTR loci (e.g. two LTRs sharing a read are assigned 0.5 read each). Second, multiple mapping reads were assigned to a single locus and the highest possible density of reads for a single locus was recorded. Similarly to the HybMap probe assignment procedure, these two alternatives reflect the two extremes of possible LTR transcription; all LTRs being transcribed at a similar level, or a minimum of LTR loci being responsible for all the detected transcription.

**Figure 5:**
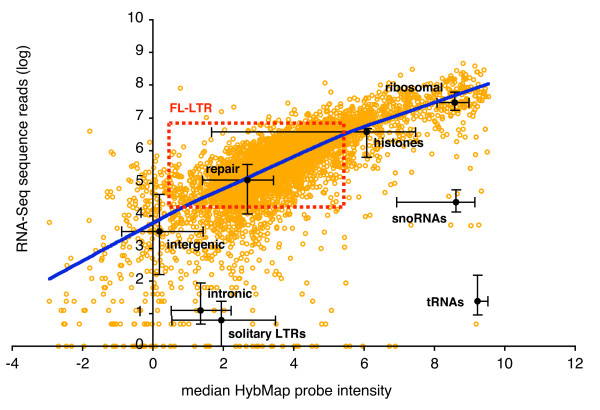
**Comparison of RNA-Seq and HybMap data**. Scatter plot between RNA-Seq abundance and median HybMap intensities for all *S. pombe *protein-coding genes (orange circles). RNA-Seq abundance calculated as log(reads per loci). HybMap values defined as the median intensity of all probes mapping to a given feature on the forward strand. Lowess curve (blue line) for all protein-coding genes is plotted using two thirds of the observations as smoothing span. Black circles denote median values for genomic features as indicated on the figure (forward strand probes only), with error bars corresponding to 25 and 75 percentiles. The possible range of transcriptional activity from full-length LTR retrotransposons is outlined as a red rectangle with dotted lines (see main text for an explanation).

We then compared the transcriptional levels of all protein-coding genes as indicated by RNA-Seq and HybMap. One initial observation is that calculating the density of RNA-Seq reads introduces a bias against longer genes (Additional file 2; Figure S7). This is of particular concern if we want to analyse transcription from full-length LTR retrotransposons, which with an average genomic length of 4912 base pairs are among the 2 percent longest genes in *S. pomb*e (not shown). We therefore used the absolute number of RNA-Seq reads per gene loci (log_10 _transformed), which show a clear correlation with HybMap levels (Figure 5), in an unbiased fashion with regards to length (Additional file 2; Figure S7). In Figure 5, the transcriptional activity from full-length LTR elements are shown as a rectangular space, in which the horizontal boundaries are determined by the median HybMap probe intensity assuming that all LTRs are transcribed equally (left side of rectangle), or assuming that the intensity for each probe stems from a minimal number of transcriptionally active loci (right side). The vertical boundaries are similarly defined for RNA-Seq read coverage. The potential range of full-length LTR transcription follows the general distribution of protein-coding genes (Figure 5). The transcriptional activity from solitary LTR sequences, as well as snoRNAs and tRNAs are underestimated in the RNA-Seq analysis compared to the HybMap approach. Lower signals from these presumably non-polyadenylated transcripts would be expected from the RNA-Seq approach (see above).

In summary, we find that the RNA-Seq data supports the finding of transcriptional activity from full-length LTR retrotransposons, and the notion that these transcripts are polyadenylated.

### LTR transcription during meiosis

**Figure 6:**
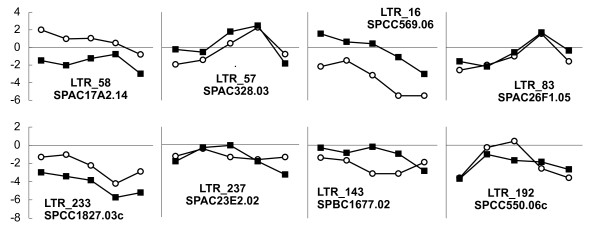
**Correlation of transcriptional activity**. RNA-Seq expression profiles for eight solitary LTRs and their neighbouring protein-coding genes across five meiosis stages, M1-M5. For each stage, the log2 ratio between stage read coverage and read coverage in growth phase is plotted (the five points hence corresponding to the five meiosis stages). Genes are shown as black squares, LTRs as open circles. Gene names and LTR numbers (Additional file 1) are provided. Log2 ratio values are shown on leftmost axes only, but are identical throughout each row.

The RNA-Seq study includes time series samples during meiosis, and we attempted to evaluate the association between LTR transcription and the transcription of neighbouring protein-coding genes during meiosis. We selected 8 LTRs with the highest uniquely mapping sequence read coverage and residing within 1000 bp upstream of a protein-coding gene (LTR sequences identified in Additional file 1; Table S2). For each meiosis stage, we calculated the ratio between read density at meiosis and read density at growth phase, and compared these to the same ratios for the neighbouring protein-coding genes. This approach allowed us to directly compare read levels without adjusting for sequence lengths and uniqueness. As seen from Figure 6, an apparent correlation is observed between some LTRs and their neighbouring genes. The scarcity of points makes correlation analysis problematic. Yet, we have attempted to assess the significance of the correlations using two different approaches. First, we concatenated the 8 time series for LTRs and for genes and calculated a product moment correlation coefficient of 0.425, which is significant below the 0.01 confidence level. Second, we produced 10.000 permutated sets of the 8 LTR expression profiles, in which the time points were shuffled independently and calculated correlation coefficients between the permutated LTR sets and the real gene sets (see Methods). We then compared the median of the product moment correlation coefficients from the real data (median = 0.802) to the distribution of medians from the permutated data. Of the 10.000 permutated sets, only a single set exceeded the real median, corresponding to a two-tailed significance level below 2 × 10^-4 ^(Additional file 2; Figure S8).

**Figure 7:**
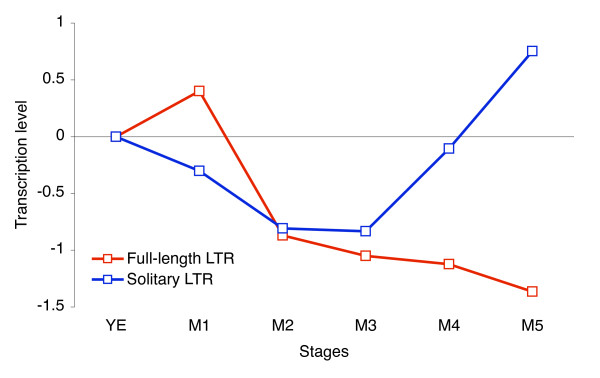
**Transcriptional activity through meiosis**. RNA-Seq expression profiles for solitary LTR sequences and full-length LTR retrotransposons across five meiotic stages. The relative level of transcription was calculated as number of reads mapping exclusively to LTR sequences divided by the total number of reads from the stage. For meiotic stages M1-M5, the log2 ratio between the given stage and the growth stage (YE) is plotted.

Comparing the transcriptional activity of full-length LTR retrotransposons to solitary LTRs during meiosis shows no apparent correlation (Figure 7), supporting the notion that these two groups of sequences behave transcriptionally distinct. It has recently been reported that CENP-B homologues of *S. pombe *are negatively regulating transcription by binding to LTR sequences (both solitary LTRs and LTRs flanking full-length elements) and facilitating chromatin modification [27]. Additionally, other apparently distinct protein pathways control LTR transcription in *S. pombe *[28,29], although RNA interference appears only to have a limited role [30]. When comparing the meiosis expression profiles of proteins involved in transcription repression to the profiles for LTR sequences, no conclusive pattern of inverse correlation between repressor genes and LTR sequences is apparent (Additional file 2; Figure S9). On the other hand, the different transcription patterns of solitary and full-length LTRs and the indications from our analysis that large differences in transcriptional activity exist between LTR loci suggest that LTR transcription is not solely determined by a global mechanism exerting its effect across the entire genome.

## Conclusions

Our analysis of two independent transcriptome data sets clearly indicates that LTR retrotransposons are actively transcribed during growth phase, and that the retrotransposon transcripts are polyadenylated. When considering the evolutionary dynamics of *S. pombe *LTR elements, Kelly and Levin [21] speculated if only a few of the complete elements were responsible for majority of transposition events (by necessity preceded by transcriptional activity) as has been observed in *Saccharomyces cerevisiae *[31]. Although we cannot conclude anything on the transcriptional levels of individual loci, our data strongly suggest that a minority of loci contribute the majority of the transcriptional output from LTR retrotransposons.

Assuming that high levels of retrotransposition are detrimental to the host, selection will favour regulation of retrotransposition. Such regulation may either take place pre-transcriptionally (e.g. methylation or chromatin condensation) or post-transcriptionally (e.g. degradation of transcripts). The observed transcription of S. pombe LTR retrotransposons suggests that post-transcriptional mechanisms are the predominant type of regulation during regular growth phase. The relatively high levels of transcriptional activity antisense to full-length LTR retrotransposons (Figures 2 and 3) may potentially be part of such post-transcriptional regulation pathways. The targeting of LTR insertion upstream of genes [23,24] (which can be viewed as a means to minimize the deleterious effects of retrotransposition in a compact genome [32]) could limit the potential of regulating retrotransposition pre-transcriptionally in S. pombe, as any interference with transcriptional regulation presumably would also affect neighbouring protein-coding genes.

From the HybMap data transcriptional activity is detectable from both strands of solitary LTR sequences, and the transcription appears to be confined to the LTR sequences themselves. Analysis of the RNA-Seq data suggests that transcription of solitary LTRs upstream of genes is correlated with the transcriptional activity of the neighbouring genes. One might speculate that the presence of transcripts from both strands of the solitary LTRs could generate double-stranded RNAs, similar to the double-stranded RNAs involved in heterochromatin formation in *S. pombe *[33,34]. However, this would suggest a negative correlation between the transcriptional activity of genes and LTR sequences. The observed positive correlation rather suggests that the LTR transcripts represent transcription facilitated by a physical association with actively transcribed genes, in parallel to the observed co-expression of linked genes [35].

Our analysis indicates that a clear distinction exists between solitary LTR sequences and full-length LTR elements in terms of transcriptional activity. Transcription of full-length LTR retrotransposons is for the most part derived from the forward strand and transcripts are polyadenylated. In contrast, solitary LTR transcription is found from both strands, with transcripts showing no signs of polyadenylation. Additionally, divergent expression levels during meiosis are observed between full-length LTR retrotransposons and solitary LTR sequences. In HIV retroviruses, a number of transcription factors bind the internal retrotransposon downstream of the 5' LTR [36]. It is possible that the absence of such binding sites around solitary LTRs contributes to their changed transcription patterns. Similarly, in a range of retrovirus LTRs, polyadenylation signals are present downstream of the transcription start sites, but a minimum distance is required for polyadenylation to take place [37], so that transcription is initiated in the 5' LTR and polyadenylation only in the 3' LTR of full-length retroviruses. Certainly, this would preclude polyadenylation of transcripts initiated within solitary LTR sequences. Similar to the solitary LTR sequences, levels of transcriptional activity appear to be relatively similar from both strands of the LTRs from full-length retrotransposons. One possible scenario is therefore that - compared to full-length retrotransposons - the solitary LTR sequences have changed their transcriptional patterns due to loss of regulatory motifs, and lost the ability to generate polyadenylated transcripts, but have retained the ability to generate transcription from both strands (at equal, albeit relatively low levels).

Based on our analysis, we conclude that the application of large-scale transcriptome data allows the elucidation of retrotransposon transcriptional activity, but that the resolution by which transcription can be assigned to specific retrotransposon loci is still limited. A recent RNA-Seq approach revealed an up-regulation of transposons in methylation-defective *Arabidopsis *mutants [38]. Additionally, mapping of capped sequence reads demonstrate wide-spread, regulated transcription initiated with mammalian retrotransposons [4]. Hence, novel transcriptome analysis techniques will inevitably shed light on tissue-specific (if applicable) and temporal expression patterns of retrotransposons facilitating an assessment of the dynamics and immediate impact of these long-term residents of eukaryotic genomes.

## Methods

### Solitary LTR and full-length LTR retrotransposon alignments

LTR sequence coordinates were extracted from the *S. pombe *genome annotation files (version 16-08-2008) downloaded from the Sanger http://www.sanger.ac.uk ftp site. Full-length LTR retrotransposons were retrieved and aligned using MUSCLE [39]. To construct the set of relatively similar solitary LTRs, all LTR sequences not being part of full-length LTR retrotransposons were aligned, and all pair-wise identity scores were recorded. LTRs were then clustered if their level of identity exceeded a certain threshold, and collapsed with other clusters if any member of one cluster had high enough similarity to any member of another cluster. By observing the changes in cluster sizes for different similarity thresholds, 70% identity was chosen as cut-off value. The members of the largest cluster were then re-aligned separately and subsequently trimmed manually removing low-similarity flanking sequences. The alignment of solitary LTR sequences is provided as Additional file 2; Figure S10 and the LTR sequences are marked as 'Context solitary LTRs' in Additional file 1; Table S2.

### Retrieval and mapping of sequence reads and probes

For RNA-Seq data, fastq files were downloaded from ArrayExpress http://www.ebi.ac.uk/microarray-as/ae/, accession number E-MTAB-5. Reads with ambiguous calls (Ns) were omitted. Reads were then mapped onto the LTRs sets (solitary and full-length) as well as the other selected genomic features using the Tagger software [40]. Only perfect matches were considered. Reads mapping to any set of genomic features were then mapped against the remaining genome, and reads and probes not mapping exclusively (for solitary LTRs and full-length LTR retrotransposons) or uniquely (all other genomic features) within a sequence set were excluded from the analysis.

HybMap data were downloaded from the Gene Expression Omnibus (GEO) at NCBI http://www.ncbi.nlm.nih.gov/, accession number GSE11619. Probes were mapped and filtered similarly to RNA-Seq sequence reads (although only probes mapping uniquely to solitary LTRs were considered), and their signal intensities normalised by a 'baseline' of intergenic values [26] were extracted. The total number of sequence reads and probes mapping to LTRs are shown in Additional file 1; Table S1. Mapping probes to LTR alignments were done by collecting the probes mapping exclusively to LTR sequences included in the alignment. The first instance of a mapping to an LTR sequence was selected, and the midpoint of the mapping position on the sequence was transferred to the corresponding column position in the alignment.

### Genomic features

Genomic coordinates for histone, ribosomal, repair and tRNA genes, as well as introns were retrieved from the genome annotation. A set of H/ACA box snoRNA sequences were collected from supplementary Table S2 in reference [41]. Genomic coordinates for snoRNAs were then established from genomic BLAST searches using stand-alone WUBLAST (now AB-BLAST http://www.advbiocomp.com/). SnoRNAs, histone, ribosomal and repair genes are listed as Additional file 1; Tables S3-S6. Intergenic sequences are defined as genomic sequence residing between protein-coding genes, tRNAs, snoRNAs and LTR sequences. To avoid putative untranslated regions (UTRs), 240 base pairs flanking both sides of protein-coding genes were omitted from the intergenic sequences (the size of 240 being adopted directly from the Cairns lab HybMap study [26]). Only intronic and intergenic sequences of at least 100 bp in size were included in the reference sets.

### Variance and correlation analyses

To assess if transcriptional activity from solitary LTR sequences were randomly distributed between the sequences, the 649 forward and 649 reverse probes mapping uniquely to 177 LTR solitary sequences were collected (forward and reverse probes analysed separately). For each LTR sequence, the average signal intensity was calculated, and the observed variance between LTR signals was recorded (the real variance). The probes were then shuffled between LTR sequences, so that each LTR sequence was assigned the same number of probes as in the real data. For the simulated set, the average signal intensity for each LTR, and the variance between LTRs was similarly calculated (the simulated variance). The simulation procedure was repeated 10.000 times for both forward and reverse probes.

Correlation analysis of the transcriptional activity between solitary LTRs and their neighbouring genes was performed as follows: LTR sequences with high levels of uniquely mapped RNA-Seq reads were collected by filtering out LTRs with a minimum of 30 uniquely mapped reads from all stages combined, and at least 10 uniquely mapped reads from growth phase. These rather arbitrarily set thresholds resulted in the eight pairs of LTRs and protein-coding genes depicted in Figure 6. The permutated sets were constructed by randomly assembling eight sets of LTR time series by shuffling the real data while keeping time points constant. For example, all M1 time points were shuffled independently, as were time points M2, M3, M4, M5. The eight sets were then compared to the real data from protein-coding genes, eight product moment correlation coefficients were calculated, and the median of coefficients was recorded. This procedure was then repeated 10.000 times.

## Authors' contributions

TM designed and performed the analysis. TM and EW wrote the paper. Both authors read and approved the final draft.

## Supplementary Material

Additional file 1 **Supplementary Tables**. Supplementary Tables S1-S6. Click here for file

Additional file 2 **Supplementary Figures**. Supplementary Figures S1-S10. Click here for file
