# Supplementary material for: Large-scale transcriptome data reveals transcriptional activity of fission yeast LTR retrotransposons
Source: BMC Genomics. 2010 Mar 12;11:167. doi: 10.1186/1471-2164-11-167 (PMC2848245; doi:10.1186/1471-2164-11-167)
Supplement: Additional file 1 — Supplementary Tables. Supplementary Tables S1-S6. [file 1471-2164-11-167-S1.PDF]

Additional File 1

Large-Scale Transcriptome Data Reveals Transcriptional Activity of Fission Yeast LTR Retrotransposons

Tobias Mourier, Eske Willerslev

Supplementary Tables S1-S6

Table S1. Overview of data samples

RNA-Seq samples

Sample	Reads mapping exclusively to: full-length LTR	solitary LTR	Description
YE	901	817	Growth, Rich medium
M1	513	286	Meiosis, 0 hours (h)
M2	241	228	Meiosis, 1-2 h
M3	205	216	Meiosis, 3-4 h
M4	178	327	Meiosis, 5-6 h
M5	77	303	Meiosis, 7-8 h

HybMap samples

Sample	Probes mapping: exclusively to full-length LTR	uniquely to solitary LTR	Description
exp1	1032	1298	Growth, Rich medium, standard
exp2	1032	1298	Growth, Rich medium, poly(A)-enriched RNA
exp3	1032	1298	Minimal medium
exp4	1032	1298	Heat Shock
exp5	1032	1298	MMS, DNA damage

Table S2

A. Solitary LTR sequences

ID	chr	start	stop	size	Context solitary LTRs[a]	LTR/gene correlation[b]	Unique HybMap probes[c]	HybMap probes[d]	Unique RNA-Seq reads[e]	RNA-Seq reads[f]
LTR_1	1	21035	21329	295			0	11	0	1
LTR_2	1	24281	24445	165			4	4	0	0
LTR_3	1	24581	24876	296			0	0	0	0
LTR_4	1	25997	26339	343			0	0	0	0
LTR_5	1	28124	28480	357			2	15	0	4
LTR_6	1	32863	33057	195			4	5	11	11
LTR_7	1	54499	54854	356			10	10	1	1
LTR_8	1	73709	73977	269			8	8	2	2
LTR_9	1	73945	74282	338			10	10	0	0
LTR_10	1	79680	79768	89			0	0	0	0
LTR_11	1	98631	98942	312			4	7	0	1
LTR_12	1	98988	99228	241			4	7	1	1
LTR_13	1	106349	106684	336			10	10	5	5
LTR_14	1	397023	397370	348	X		6	6	0	2
LTR_15	1	402409	402782	374			12	13	1	1
LTR_16	1	446075	446342	268		X	8	8	27	27
LTR_17	1	563321	563628	308			10	10	1	1
LTR_18	1	617366	617715	350	X		6	6	5	10
LTR_19	1	669786	670131	346			10	10	0	0
LTR_20	1	1004892	1005249	358	X		4	8	4	14
LTR_21	1	1044522	1044860	339			10	10	1	1
LTR_22	1	1118434	1118790	357	X		10	10	9	10
LTR_23	1	1142619	1142965	347	X		10	10	2	2
LTR_24	1	1148611	1148794	184			4	4	32	32
LTR_25	1	1265809	1266166	358	X		0	3	0	2
LTR_26	1	1398600	1398936	337			10	10	1	1
LTR_27	1	1444293	1444655	363			10	10	0	0
LTR_32	1	1663486	1663829	344			10	10	5	5
LTR_33	1	1999638	1999964	327			10	10	8	8
LTR_34	1	2012006	2012346	341	X		4	4	11	11
LTR_35	1	2173899	2174235	337			10	10	5	5
LTR_36	1	2186786	2187129	344	X		10	10	2	2
LTR_37	1	2415505	2415860	356			12	12	1	1
LTR_38	1	2437580	2437941	362	X		2	3	0	0
LTR_39	1	2474235	2474583	349	X		0	2	0	0
LTR_40	1	2605356	2605703	348	X		10	10	3	3
LTR_41	1	2693886	2694225	340	X		10	10	1	1
LTR_42	1	2843759	2844091	333	X		10	10	0	0
LTR_43	1	2854057	2854405	349	X		0	2	0	1
LTR_46	1	2939937	2940229	293			2	2	2	2
LTR_47	1	2941944	2942286	343	X		0	0	0	0
LTR_48	1	3043430	3043787	358	X		2	2	2	2
LTR_49	1	3072450	3072805	356			10	10	8	8
LTR_50	1	3134575	3134919	345			10	10	3	3
LTR_51	1	3190954	3191311	358	X		4	4	16	16
LTR_52	1	3245333	3245683	351			10	10	1	1
LTR_53	1	3321466	3321823	358	X		4	4	0	0
LTR_56	1	3438221	3438576	356			10	10	1	1
LTR_57	1	3478114	3478322	209		X	6	6	17	17
LTR_58	1	3586218	3586423	206		X	4	4	8	8
LTR_59	1	3924228	3924580	353			12	12	4	4
LTR_60	1	3959530	3959668	139			4	4	2	2
LTR_61	1	3995538	3995887	350			10	10	2	2
LTR_66	1	4140539	4140896	358	X		4	4	2	2
LTR_67	1	4287575	4287933	359			10	10	1	1
LTR_68	1	4294232	4294523	292			8	8	15	15
LTR_69	1	4524914	4525142	229			0	6	0	8
LTR_70	1	4525245	4525599	355			0	11	0	17
LTR_71	1	4525577	4525926	350			0	11	0	9
LTR_72	1	4527951	4528179	229			0	6	0	3
LTR_73	1	4528282	4528636	355			0	11	0	14
LTR_74	1	4528614	4528963	350			0	11	0	4
LTR_75	1	4619245	4619544	300			8	8	0	1
LTR_76	1	4897509	4897866	358	X		2	3	0	2
LTR_77	1	4910978	4911115	138			4	4	6	6
LTR_78	1	4919147	4919506	360			10	10	1	1
LTR_79	1	4939621	4939972	352			8	11	0	2
LTR_80	1	4941720	4942068	349	X		0	0	0	0
LTR_81	1	5021560	5021908	349	X		6	6	0	0
LTR_82	1	5066415	5066762	348			12	12	0	0
LTR_83	1	5175815	5176226	412	X	X	4	5	9	13
LTR_87	1	5200296	5200657	362			10	10	8	8
LTR_88	1	5325149	5325497	349	X		2	2	0	0
LTR_89	1	5496305	5496655	351			10	10	19	19
LTR_90	1	5541543	5541852	310			10	10	0	0
LTR_91	1	5558866	5559091	226			6	6	2	2
LTR_92	1	5570061	5570383	323			2	3	0	0
LTR_93	2	997	1215	219			0	0	0	0
LTR_94	2	1296	1591	296			0	0	0	0
LTR_95	2	2756	3054	299			0	0	0	0
LTR_96	2	4835	5191	357			0	3	0	0
LTR_97	2	57151	57483	333			10	10	0	0
LTR_98	2	57604	57821	218			6	6	0	0
LTR_99	2	63206	63538	333			10	10	3	7
LTR_100	2	93517	93860	344			0	21	0	3
LTR_101	2	96276	96619	344			0	0	0	0
LTR_102	2	99035	99378	344			0	0	0	0
LTR_103	2	101794	102137	344			0	21	0	2
LTR_104	2	136385	136555	171			4	4	0	0
LTR_105	2	458478	458824	347			10	10	2	2
LTR_106	2	593824	594176	353			10	10	0	0
LTR_107	2	676281	676634	354	X		2	3	0	2
LTR_108	2	739738	740096	359	X		4	5	0	2
LTR_109	2	846705	847051	347	X		8	11	1	4
LTR_110	2	942476	942820	345			10	10	0	0
LTR_111	2	1034420	1034731	312			10	10	10	10
LTR_112	2	1073869	1074074	206			4	4	5	5
LTR_113	2	1079189	1079526	338			12	12	7	7
LTR_114	2	1120415	1120670	256			8	8	7	7
LTR_115	2	1532619	1532951	333			10	10	45	45
LTR_116	2	1805772	1806127	356			4	14	0	1
LTR_119	2	1826267	1826606	340			2	9	0	0
LTR_122	2	2012305	2012648	344			6	8	1	1
LTR_123	2	2080929	2081276	348	X		4	9	0	0
LTR_124	2	2091487	2091720	234			2	7	0	3
LTR_125	2	2163463	2163810	348	X		4	5	0	0
LTR_126	2	2339945	2340297	353	X		10	11	6	7
LTR_127	2	2380161	2380508	348	X		2	5	1	1
LTR_128	2	2425971	2426336	366	X		0	1	0	0
LTR_129	2	2636814	2637157	344	X		2	2	0	0
LTR_130	2	2736508	2736743	236			6	6	2	2
LTR_131	2	3105823	3106180	358	X		2	5	0	4
LTR_132	2	3136259	3136520	262			8	8	1	1
LTR_133	2	3168963	3169272	310			8	8	0	0
LTR_134	2	3282860	3283208	349	X		4	7	0	0
LTR_135	2	3474256	3474555	300			10	10	3	3
LTR_136	2	3489303	3489651	349	X		0	0	0	0
LTR_137	2	3489848	3490095	248			4	5	0	0
LTR_138	2	3489943	3490295	353			8	9	0	0
LTR_139	2	3490384	3490610	227			6	6	1	1
LTR_140	2	3659043	3659390	348	X		12	12	1	1
LTR_141	2	3676727	3677069	343			10	10	4	4
LTR_142	2	3714033	3714382	350			10	10	2	2
LTR_143	2	3934659	3934738	80		X	2	2	18	18
LTR_144	2	4047404	4047754	351	X		10	11	0	2
LTR_145	2	4160955	4161260	306			2	5	2	3
LTR_146	2	4217977	4218323	347			10	10	1	2
LTR_147	2	4231522	4231869	348			10	10	3	3
LTR_148	2	4296668	4297025	358	X		8	10	2	2
LTR_149	2	4409793	4410139	347			10	10	0	0
LTR_152	2	4436890	4437243	354			10	10	1	1
LTR_153	2	4437498	4437846	349	X		8	9	0	0
LTR_154	2	4437850	4438205	356	X		12	12	0	0
LTR_155	2	4474977	4475138	162			0	0	0	0
LTR_156	2	4481108	4481445	338			10	10	10	10
LTR_157	2	4481502	4481842	341	X		10	10	0	1
LTR_158	2	4481838	4482117	280			8	8	2	2
LTR_159	2	4501568	4501763	196			2	3	2	5
LTR_160	2	4507733	4508064	332			0	14	0	3
LTR_161	2	4514859	4515181	323			0	12	0	4
LTR_162	3	26343	26675	333			0	10	0	0
LTR_163	3	31728	31925	198			6	6	1	1
LTR_164	3	43111	43459	349	X		0	1	0	1
LTR_165	3	49206	49550	345			10	10	9	9

A. Solitary LTR sequences (cont.)

ID	chr	start	stop	size	Context solitary LTRs[a]	LTR/gene correlation[b]	Unique HybMap probes[c]	HybMap probes[d]	Unique RNA-Seq reads[e]	RNA-Seq reads[f]
LTR_166	3	108555	108863	309			2	9	4	9
LTR_167	3	111397	111560	164			4	4	0	0
LTR_168	3	114382	114730	349	x		2	4	0	0
LTR_169	3	187086	187449	364	x		0	1	3	3
LTR_170	3	218342	218642	301			10	10	5	5
LTR_171	3	222987	223246	260			6	6	1	1
LTR_172	3	254350	254629	280			8	8	33	33
LTR_173	3	257183	257531	349	x		4	6	1	1
LTR_174	3	286728	287053	326			10	10	2	2
LTR_175	3	298547	298890	344	x		8	11	0	0
LTR_176	3	298890	299217	328			10	10	13	13
LTR_177	3	381641	382003	363	x		2	5	3	4
LTR_178	3	486575	486925	351			12	12	1	1
LTR_179	3	489072	489396	325	x		2	5	0	1
LTR_180	3	489375	489697	323	x		8	9	0	1
LTR_181	3	499951	500308	358	x		4	8	0	0
LTR_182	3	500370	500717	348			10	10	1	1
LTR_183	3	552825	552963	139			2	2	4	4
LTR_184	3	614910	615258	349			10	10	2	2
LTR_185	3	701782	702130	349	x		0	0	0	0
LTR_186	3	702136	702501	366			8	8	0	0
LTR_189	3	954537	954860	324			8	11	0	0
LTR_190	3	958713	958931	219			6	6	0	0
LTR_191	3	1176250	1176592	343			10	10	1	1
LTR_192	3	1195985	1196059	75		x	0	0	8	8
LTR_193	3	1207147	1207503	357	x		10	10	2	5
LTR_194	3	1296337	1296666	330			10	10	3	3
LTR_195	3	1400870	1401218	349	x		4	7	0	1
LTR_196	3	1466109	1466416	308	x		0	5	0	2
LTR_197	3	1466447	1466627	181			4	4	0	0
LTR_198	3	1496536	1496879	344			10	10	0	0
LTR_199	3	1530150	1530498	349	x		2	7	0	0
LTR_200	3	1574724	1575071	348	x		8	10	0	0
LTR_201	3	1579176	1579526	351			8	9	0	2
LTR_202	3	1581982	1582318	337	x		2	9	0	5
LTR_203	3	1629525	1629874	350			10	10	2	2
LTR_204	3	1632188	1632545	358	x		8	13	0	9
LTR_205	3	1684578	1684917	340			10	10	2	2
LTR_206	3	1716281	1716629	349	x		0	0	0	0
LTR_207	3	1738418	1738761	344			12	12	1	1
LTR_208	3	1740961	1741307	347			10	10	4	4
LTR_209	3	1808837	1809175	339			10	10	2	2
LTR_210	3	1862027	1862384	358	x		8	8	6	7
LTR_211	3	1894223	1894553	331			6	11	6	10
LTR_212	3	1933057	1933398	342			10	10	3	3
LTR_213	3	2017004	2017345	342			10	10	0	0
LTR_214	3	2021939	2022290	352			12	12	2	2
LTR_215	3	2040788	2041141	354	x		6	8	1	1
LTR_216	3	2063791	2064140	350			10	11	1	3
LTR_217	3	2083919	2084275	357	x		2	5	1	9
LTR_218	3	2108288	2108631	344			12	12	4	4
LTR_219	3	2110854	2111180	327			10	10	0	0
LTR_220	3	2119789	2120137	349	x		8	9	1	1
LTR_221	3	2145096	2145433	338	x		6	11	0	0
LTR_222	3	2147834	2148179	346			12	12	0	0
LTR_223	3	2148154	2148502	349	x		0	0	0	1
LTR_224	3	2158747	2159089	343	x		2	5	0	2
LTR_225	3	2180475	2180832	358	x		2	4	0	1
LTR_226	3	2210258	2210588	331			10	10	4	4
LTR_227	3	2210640	2210988	349	x		4	5	0	1
LTR_228	3	2220430	2220754	325			10	10	0	0
LTR_229	3	2222680	2223022	343			10	10	6	6
LTR_230	3	2230367	2230688	322	x		10	10	1	1
LTR_233	3	2380886	2381185	300		x	8	8	30	30
LTR_234	3	2400868	2401095	228			6	6	0	0
LTR_235	3	2405874	2406228	355	x		10	10	2	2
LTR_236	3	2418126	2418384	259			6	6	4	4
LTR_237	3	2422329	2422687	359		x	12	12	37	40
LTR_238	3	2432243	2432411	169			4	4	2	4
LTR_239	3	2437467	2437780	314			0	10	0	0

B. Full-length LTR retrotransposons

Retrotransposon	chr	start	stop	LTR 1 ID	LTR 2 ID	size	Gene	Pseudogene	Unique HybMap probes[c]	HybMap probes[d]	Unique RNA-Seq reads[e]	RNA-Seq reads[f]
A	1	1465332	1470247	LTR_28	LTR_29	4916	SPAC9.04		0	988	0	893
B	1	1563817	1568732	LTR_31	LTR_30	4916	SPAC1705.01c		2	954	8	863
C	1	2926805	2931720	LTR_45	LTR_44	4916	SPAC2E1P3.03c		0	1032	0	895
D	1	3361148	3366062	LTR_55	LTR_54	4915	SPAC26A3.13c		0	986	0	892
E	1	3996024	4000939	LTR_63	LTR_62	4916	SPAPB15E9.03c		0	1032	0	895
F	1	4021977	4026892	LTR_64	LTR_65	4916	SPAC27E2.08		0	1032	0	895
G	1	5190745	5195667	LTR_85	LTR_84	4923	SPAC13D1.01c		0	986	0	923
H	1	5195312	5200227	LTR_86	LTR_85	4916	SPAC19D5.09c		0	986	0	914
I	2	1812349	1817262	LTR_118	LTR_117	4914	SPBC9B6.02c		2	988	0	892
J	2	1964875	1969789	LTR_120	LTR_121	4915	SPBC1E8.04	x	6	948	2	874
K	2	4414197	4419057	LTR_150	LTR_151	4861	SPBC8E4.11c		32	984	6	910
L	3	777734	782649	LTR_188	LTR_187	4916	SPCC1020.14		0	1032	0	895
M	3	2319921	2324835	LTR_232	LTR_231	4915	SPCC1494.11c	x	2	960	0	885

Table S2. LTR sequences

a) LTR sequences included in the aligned solitary LTRs with analysed context indicated by 'x'
b) LTR sequences included in the correlation analysis of solitary LTRs and neighbouring genes indicated by 'x'
c) HybMap probes mapping uniquely to sequence
d) LTR-specific HybMap probes mapping to sequence
e) RNA-Seq reads from growth phase mapping uniquely to sequence
f) LTR-specific RNA-Seq reads from growth stage mapping to sequence

Table S3. H/ACA box snoRNA sequences used in the study

ID	chromosome	start	end	strand
snR97	chr1	170709	170856	1
Sp16-56	chr1	483162	483316	-1
Sp17-17	chr1	2265681	2265848	-1
snR98	chr1	2330436	2330647	-1
snR101	chr1	2789709	2789981	1
snR91	chr1	3316466	3316609	-1
Sp17-10	chr1	3394878	3395062	1
Sp12-55	chr1	4173319	4173463	1
snR90	chr1	4937237	4937394	-1
snR3	chr2	884166	884332	-1
snR95	chr2	1468185	1468406	-1
snR42	chr2	1917406	1917613	1
snR36	chr2	1957286	1957449	-1
snR92	chr2	2121215	2121358	1
snR5	chr2	2266027	2266169	-1
snR33	chr2	2447904	2448048	-1
snR93	chr2	3172781	3172923	1
snR100	chr2	3393616	3393802	1
snR99	chr3	583416	583605	1
snR94	chr3	923877	924086	1
snR10	chr3	992436	992602	-1
snR46	chr3	1717920	1718087	-1
Sp14-61	chr3	2248862	2249088	-1

Table S4. Histone protein genes used in the study

ID	description
SPAC19G12.06c	H2A beta
SPAC1834.03c	H4 h4.1
SPAC1834.04	H3 h3.1
SPBC800.13	H4 variant
SPBC8D2.03c	H4 h4.2
SPBC8D2.04	H3 h3.2
SPBC11B10.10c	H2A variant Pht1
SPBC1105.11c	H3 h3.3
SPBC1105.12	H4 h4.3
SPBC1105.17	H3 variant CENP-A
SPCC622.08c	H2A alpha
SPCC622.09	H2B alpha Htb1

Table S5. Ribosomal protein genes used in the study

ID	description
SPBC16G5.14c	40S ribosomal protein S3
SPAC22E12.13c	60S ribosomal protein L24-3 (L30)
SPAPB17E12.05	60S ribosomal protein L37
SPCC330.14c	60S ribosomal protein L24
SPAC5D6.01	40S ribosomal protein S15a
SPAP7G5.05	60S ribosomal protein L10
SPAPB17E12.13	60S ribosomal protein L18
SPBC29A3.04	60S ribosomal protein L7a (L8)
SPCC74.05	60S ribosomal protein L27
SPBC29A3.12	40S ribosomal protein S9
SPBP8B7.03c	60S ribosomal protein L2
SPBC1685.09	40S ribosomal protein S29
SPAC24H6.07	40S ribosomal protein S9
SPBC19G7.03c	40S ribosomal protein S30
SPBC1685.10	40S ribosomal protein S27
SPAC13G6.02c	40S ribosomal protein S3a
SPAC521.05	40S ribosomal protein S8
SPAC3G9.03	60S ribosomal protein L23
SPBC16C6.11	60S ribosomal protein L32
SPAC11E3.15	60S ribosomal protein L22
SPBC839.04	60S ribosomal protein L8
SPAC6G9.09c	60S ribosomal protein L24
SPAC328.10c	40S ribosomal protein S5
SPCC576.08c	40S ribosomal protein S2
SPBC365.03c	60S ribosomal protein L21
SPAC13G6.07c	40S ribosomal protein S6
SPAC3H5.07	60S ribosomal protein L7
SPAC1071.07c	40S ribosomal protein S15
SPAC3H5.10	60S ribosomal protein L32
SPAC890.08	60S ribosomal protein L31
SPAC144.11	40S ribosomal protein S11
SPBC1711.06	60S ribosomal protein L2
SPAC23A1.08c	60S ribosomal protein L34
SPCC1259.01c	40S ribosomal protein S18
SPAC31G5.03	40S ribosomal protein S11
SPBC1921.01c	60S ribosomal protein L35a
SPBC2F12.07c	60S ribosomal protein L8
SPAC959.08	60S ribosomal protein L21
SPAC959.07	40S ribosomal protein S4
SPBC649.02	40S ribosomal protein S19
SPAC19B12.04	40S ribosomal protein S30
SPBC21C3.13	40S ribosomal protein S19
SPCC613.05c	60S ribosomal protein L35
SPBC16D10.11c	40S ribosomal protein S18
SPAPB8E5.06c	60S ribosomal protein L3
SPAC1783.08c	60S ribosomal protein L15b
SPCC576.09	40S ribosomal protein S20
SPCC576.11	60S ribosomal protein L15
SPCC1682.14	60S ribosomal protein L19
SPBC11C11.09c	60S ribosomal protein L5
SPBC2F12.04	60S ribosomal protein L17
SPCC24B10.09	40S ribosomal protein S17
SPBC776.01	60S ribosomal protein L29
SPBC776.11	60S ribosomal protein L27/L28
SPCC663.04	60S ribosomal protein L39
SPBC3D6.15	40S ribosomal protein S25
SPBC405.07	60S ribosomal protein L36
SPAPJ698.02c	40S ribosomal protein S0B
SPAC25G10.06	40S ribosomal protein S28
SPAC664.04c	40S ribosomal protein S16
SPBP22H7.08	40S ribosomal protein S10
SPBC577.02	60S ribosomal protein L38

ID	description
SPBP4H10.13	40S ribosomal protein S23
SPAC664.05	60S ribosomal protein L13
SPAC664.06	60S ribosomal protein L7
SPCC622.18	60S ribosomal protein L6
SPBC56F2.02	60S ribosomal protein L19
SPBC18H10.13	40S ribosomal protein S14
SPBC18H10.14	40S ribosomal protein S16
SPBC685.07c	60S ribosomal protein L27
SPAC30D11.12	60S ribosomal protein L38
SPCP31B10.08c	60S ribosomal protein L35a
SPBC4F6.04	60S ribosomal protein L25
SPBC800.04c	60S ribosomal protein L37a
SPBC83.02c	60S ribosomal protein L37a
SPBC839.13c	60S ribosomal protein L13/L16
SPAPB1E7.12	40S ribosomal protein S6
SPAC694.05c	40S ribosomal protein S25
SPAC1F7.13c	60S ribosomal protein L8
SPAC3A12.10	60S ribosomal protein L20a
SPAC17G6.06	40S ribosomal protein S24
SPAC26A3.07c	60S ribosomal protein L11
SPCC285.15c	40S ribosomal protein S28
SPCC16C4.13c	60S ribosomal protein L12.1/L12A
SPAC8C9.08	40S ribosomal protein S5
SPCC1393.03	40S ribosomal protein S15
SPAC17A5.03	60S ribosomal protein L3
SPCC1183.08c	60S ribosomal protein L10a
SPBC839.05c	40S ribosomal protein S17
SPBC685.06	40S ribosomal protein S0A (p40)
SPCC5E4.07	60S ribosomal protein L27/L28
SPAC15E1.03	60S ribosomal protein L36/L42
SPAC22A12.04c	40S ribosomal protein S15a
SPBC18E5.04	60S ribosomal protein L10
SPBC18E5.06	40S ribosomal protein S21
SPCC364.03	60S ribosomal protein L17
SPAC9G1.03c	60S ribosomal protein L30
SPBC17G9.10	60S ribosomal protein L11
SPBC17G9.07	40S ribosomal protein S24
SPCC962.04	40S ribosomal protein S12
SPAC3H5.05c	40S ribosomal protein S14
SPAC23C11.02c	40S ribosomal protein S23
SPAC806.03c	40S ribosomal protein S26
SPCC613.06	60S ribosomal protein L9
SPCC1223.05c	60S ribosomal protein L37
SPAC3H5.12c	60S ribosomal protein L5
SPBC2G2.05	60S ribosomal protein L13/L16
SPBC18H10.12c	60S ribosomal protein L7
SPBC30D10.18c	60S ribosomal protein L10a
SPCC970.05	60S ribosomal protein L36
SPAC26A3.04	60S ribosomal protein L20
SPAC31G5.17c	40S ribosomal protein S10
SPBC21B10.10	40S ribosomal protein S4
SPAC23A1.11	60S ribosomal protein L13/L16
SPCC1322.15	60S ribosomal protein L34
SPBC29B5.03c	60S ribosomal protein L26
SPCC1322.11	60S ribosomal protein L23
SPBC1685.02c	40S ribosomal protein S12
SPAC3G6.13c	60S ribosomal protein L41
SPBC19F8.08	40S ribosomal protein S4
SPAC1250.05	60S ribosomal protein L30
SPBC11C11.07	60S ribosomal protein L18
SPAC2C4.16c	40S ribosomal protein S8
SPAC4G9.16c	60S ribosomal protein L9

Table S6. Repair genes used in the study

ID	description
SPAC15A10.03c	rad54
SPBC3E7.08c	rad13
SPAC13G6.01c	rad8
SPBC4F6.15c	rad23
SPAC1D4.12	rad15
SPCC1259.13	rad27
SPBC660.13c	rad11
SPCC550.13	rad35
SPAC8E11.02c	rad24
SPAC13C5.07	rad32
SPAC17A2.13c	rad25
SPAC664.07c	rad9
SPCC338.17c	rad21
SPAC3G6.06c	rad2
SPAC4C5.04	rad31
SPAC1556.01c	rad50
SPBC119.14	rad22B
SPAC1952.07	rad1
SPAC30D11.10	rad22
SPBC20F10.04c	rad62
SPAC2G11.12	rad12
SPBC216.05	rad3
SPCC5E4.06	rad18
SPAC644.14c	rad51
SPAC9E9.08	rad26
SPBC1921.02	rad60
SPCC970.01	rad20
SPAC23C4.18c	rad4
SPAC14C4.13	rad17
SPCC613.13c	rad16
SPCC1753.03c	rec7
SPAC1002.06c	rec23
SPBC32F12.02	rec14
SPBC1711.14	rec15
SPAC17A5.18c	rec25
SPBC2D10.06	rec16
SPAC25G10.04c	rec10
SPAC1952.15c	rec24
SPCC550.16c	rec11
SPBC577.05c	rec27
SPAC17A5.11	rec12
SPBC29A10.14	rec8
SPBC21B10.12	rec6
