# Supplementary material for: Large-scale transcriptome data reveals transcriptional activity of fission yeast LTR retrotransposons
Source: BMC Genomics. 2010 Mar 12;11:167. doi: 10.1186/1471-2164-11-167 (PMC2848245; doi:10.1186/1471-2164-11-167)
Supplement: Additional file 2 — Supplementary Figures. Supplementary Figures S1-S10. [file 1471-2164-11-167-S2.PDF]

Additional File 2

Large-Scale Transcriptome Data Reveals Transcriptional Activity of Fission Yeast LTR Retrotransposons

Tobias Mourier, Eske Willerslev

Supplementary Figures S1-S10

Fig. S1. **A**) HybMap probes mapping exclusively to full-length LTR retrotransposons are plotted according to their number of mappings within the LTR set. Number of probes correspond to forward strand probes only. A similar number of probes are found on the reverse strand. **B**) The five full-length LTR retrotransposons with uniquely mapping probes. **C**) HybMap probes mapped onto an alignment of full-length LTR retrotransposons. Signal intensities for forward probes (blue circles) and reverse probes (red circles) are shown on the left y-axis. The number of retrotransposon loci that each probe is mapped onto is shown as black squares on the right y-axis.

Fig. S2. The average signal intensity of HybMap probes plotted for 214 solitary LTR sequences (x-axis) and their nearest protein coding gene (y-axis). Forward and reverse probes are pooled for LTR sequences. Forward gene probes shown as blue dots, reverse probes as orange dots. Linear regression lines are shown.

Fig. S3. The distribution of variances from 10.000 permutated sets of intra-individual LTR transcriptional activities. The observed variances from the real data are shown in text box.

Fig. S4. Construction of sets of solitary LTR and their flanking sequence. Graphic depiction of the retrieval procedure of flanking sequence from 71 highly similar solitary LTR sequences. LTR sequences are aligned and the flanking sequences are appended to the alignment. Below is a chart showing the number of sequences present along the different positions (orange line) with the plateau of 71 sequences present in

the LTR alignment. The black line shows the number of probes (both forward and reverse) present in a moving 30 base pairs window.

Fig. S5. HybMap data for from alternative procedures and growth conditions as performed by Dutrow et al. (Dutrow et al. 2008). General layout as in Figure 2 (see main text), but with data derived from different samples (as listed on charts).

Fig. S6. Signal variation across gene sequences. HybMap probes are mapped onto coding sequence of histone, ribosomal and repair genes and the relative position in the sequence (0=first base in sequence, 1=last base in sequence) are plotted against the signal intensity. For the RNA-Seq reads, genes were divided into 10 bins, and the number of mapped tags are plotted. Total RNA, HybMap probes for histone (A), ribosomal (D) and repair genes (G). Poly(A)-enriched, HybMap probes for histone (B), ribosomal (E) and repair genes (H). RNA-Seq reads for histone (C), ribosomal (F) and repair genes (I). For the HybMap plots, lowess curves are plotted for both orientations (smoothing window=1/4 of all observations).

Fig. S7. Scatterplot between RNA-Seq reads and median HybMap intensities for all *S. pombe* protein-coding genes. A) RNA-Seq reads per base pair (log-transformed). B) RNA-Seq reads per genomic loci (log-transformed). Genes were grouped on the basis of the length of their coding sequence. Short genes (<1000 bp); Green circles. Intermediate genes (1000-4000 bp); Yellow circles. Long genes (>4000 bp); Red circles. For each group of genes, lowess curves are plotted using a smoothing window of two thirds of all observations.

Fig. S8. Simulated sets of LTR/gene correlation. The distribution of the median correlation coefficient from 10.000 permutated sets of LTR/nearest gene activity measures (black bars). The observed median correlation (0.802) from the real data indicated by the arrow.

Fig. S9. Transcriptional activity through meiosis. A) Similar to Figure 7 in main text, shown for comparison. B) Expression profiles for 8 protein-coding genes shown to influence LTR transcription (Hansen et al. 2005; Greenall et al. 2006; Durand-Dubief et al. 2007; Cam et al. 2008). Transcriptional activity based on RNA-Seq reads mapped using our procedure. C) Transcriptional activity converted from data obtained from The *S.pombe* Gene Expression Viewer at [http://www.bahlerlab.info/perl/SPGE/geexview] (Mata et al. 2002).

Fig. S10. Alignment of the 71 similar solitary LTR sequences used for the analysis of transcriptional activity from LTR sequences and their genomic context.

References for Supplementary Figures

Cam, H. P., K. Noma, H. Ebina, H. L. Levin, and S. I. Grewal. 2008. Host genome surveillance for retrotransposons by transposon-derived proteins. Nature **451**:431-436.

Durand-Dubief, M., I. Sinha, F. Fagerstrom-Billai, C. Bonilla, A. Wright, M. Grunstein, and K. Ekwall. 2007. Specific functions for the fission yeast Sirtuins Hst2 and Hst4 in gene regulation and retrotransposon silencing. Embo J **26**:2477-2488.

Dutrow, N., D. A. Nix, D. Holt, B. Milash, B. Dalley, E. Westbroek, T. J. Parnell, and B. R. Cairns. 2008. Dynamic transcriptome of Schizosaccharomyces pombe shown by RNA-DNA hybrid mapping. Nat Genet **40**:977-986.

Greenall, A., E. S. Williams, K. A. Martin, J. M. Palmer, J. Gray, C. Liu, and S. K. Whitehall. 2006. Hip3 Interacts with the HIRA Proteins Hip1 and Slm9 and Is Required for Transcriptional Silencing and Accurate Chromosome Segregation. J. Biol. Chem. **281**:8732-8739.

Hansen, K. R., G. Burns, J. Mata, T. A. Volpe, R. A. Martienssen, J. Bahler, and G. Thon. 2005. Global Effects on Gene Expression in Fission Yeast by Silencing and RNA Interference Machineries. Mol. Cell. Biol. **25**:590-601.

Mata, J., R. Lyne, G. Burns, and J. Bahler. 2002. The transcriptional program of meiosis and sporulation in fission yeast. Nat Genet **32**:143-147.

Figure S1

A

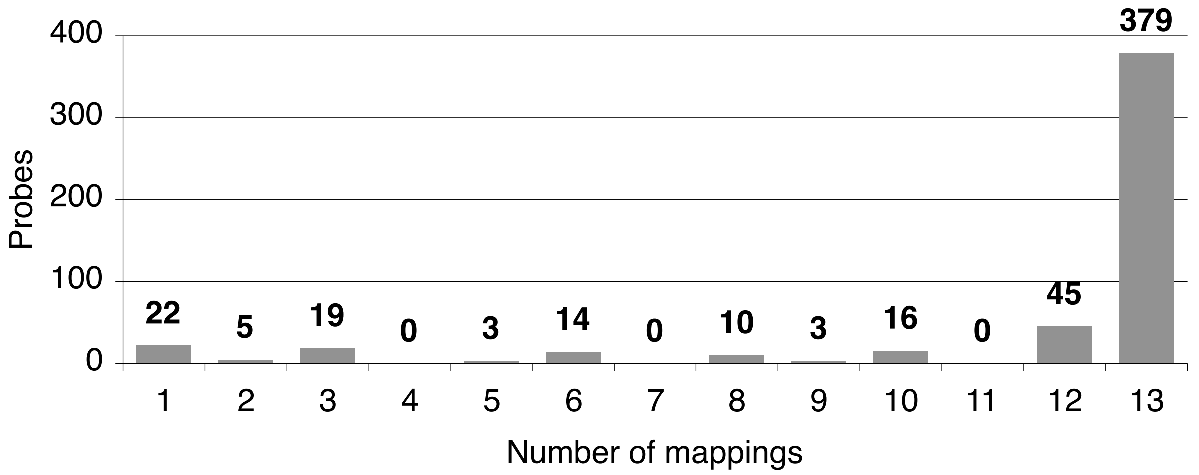

B

ID	GeneDB	coords	unique probes
LTR_B	SPAC1705.01c	chr1:1563817-1568732	1
LTR_I	SPBC9B6.02c	chr2:1812349-1817262	1
LTR_J*	SPBC1E8.04	chr2:1964875-1969789	3
LTR_K	SPBC8E4.11c	chr2:4414197-4419057	16
LTR_M*	SPCC1494.11c	chr3:2319921-2324835	1

*) Pseudogenes
Our IDs as in TableS2

C

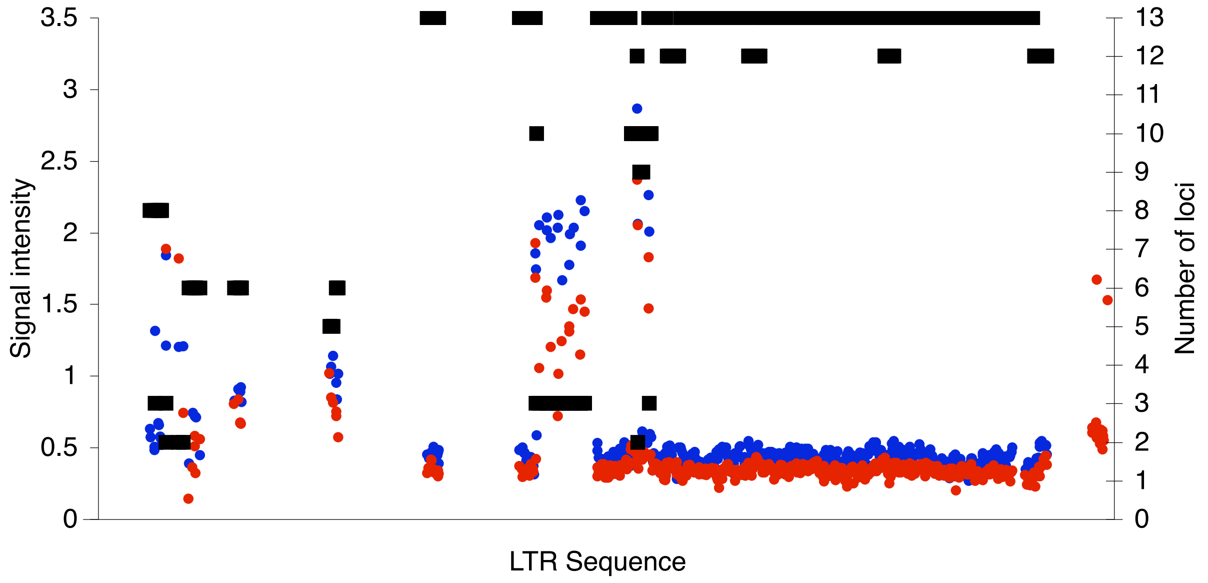

Figure S2

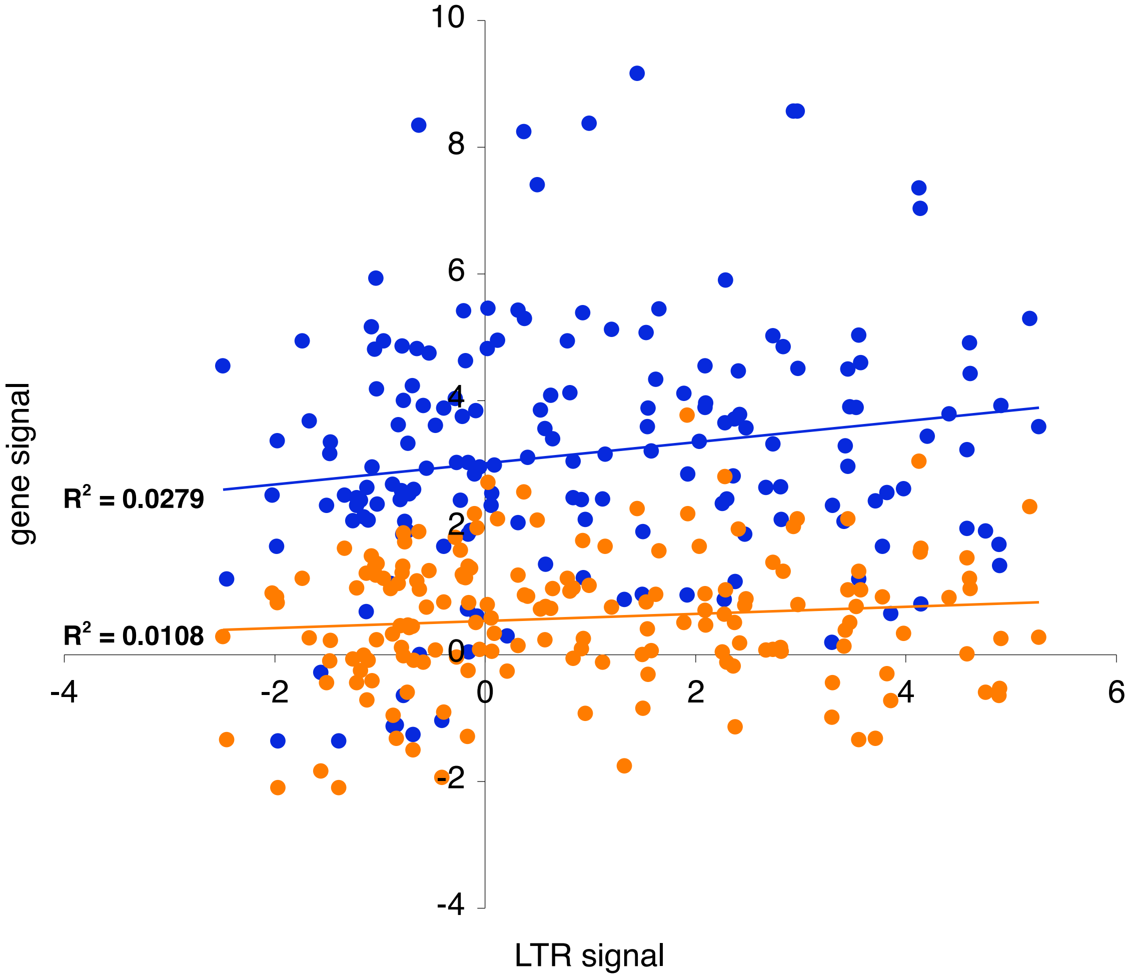

Figure S3

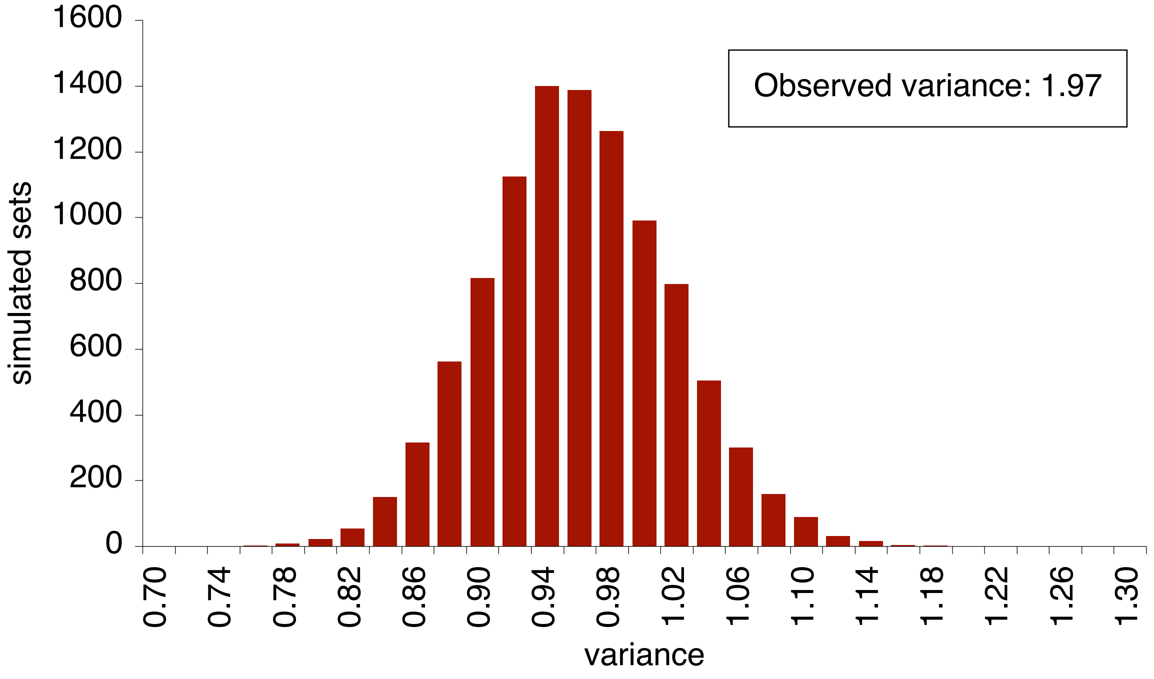

Observed variance: 1.97
simulated sets
1600
1400
1200
1000
800
600
400
200
0
0.70
0.74
0.78
0.82
0.86
0.90
0.94
0.98
1.02
1.06
1.10
1.14
1.18
1.22
1.26
1.30
variance

Figure S4

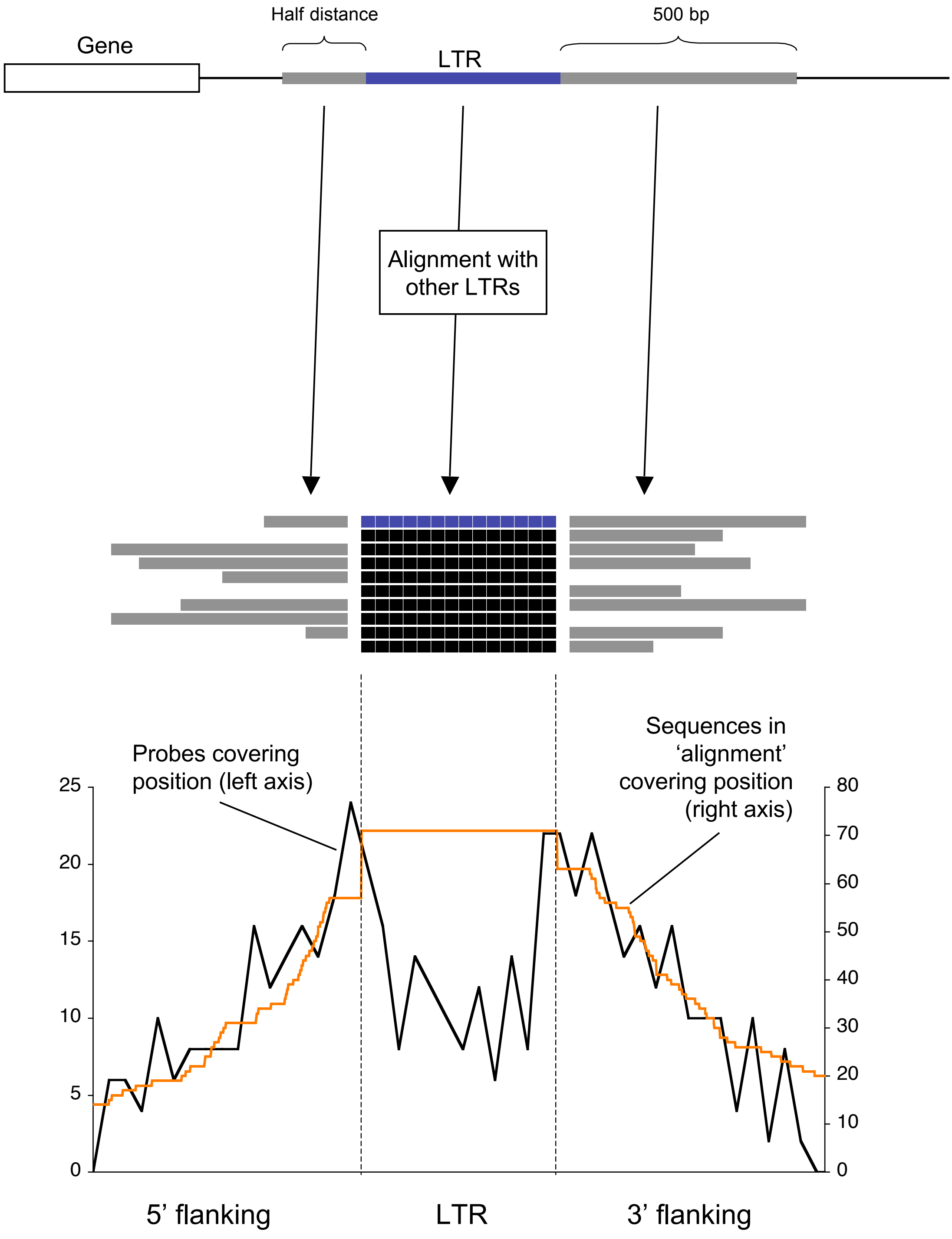

Half distance
500 bp
Gene
LTR
Alignment with other LTRs
Probes covering position (left axis)
Sequences in 'alignment' covering position (right axis)
25
20
15
10
5
0
80
70
60
50
40
30
20
10
0
5' flanking
LTR
3' flanking

Figure S5

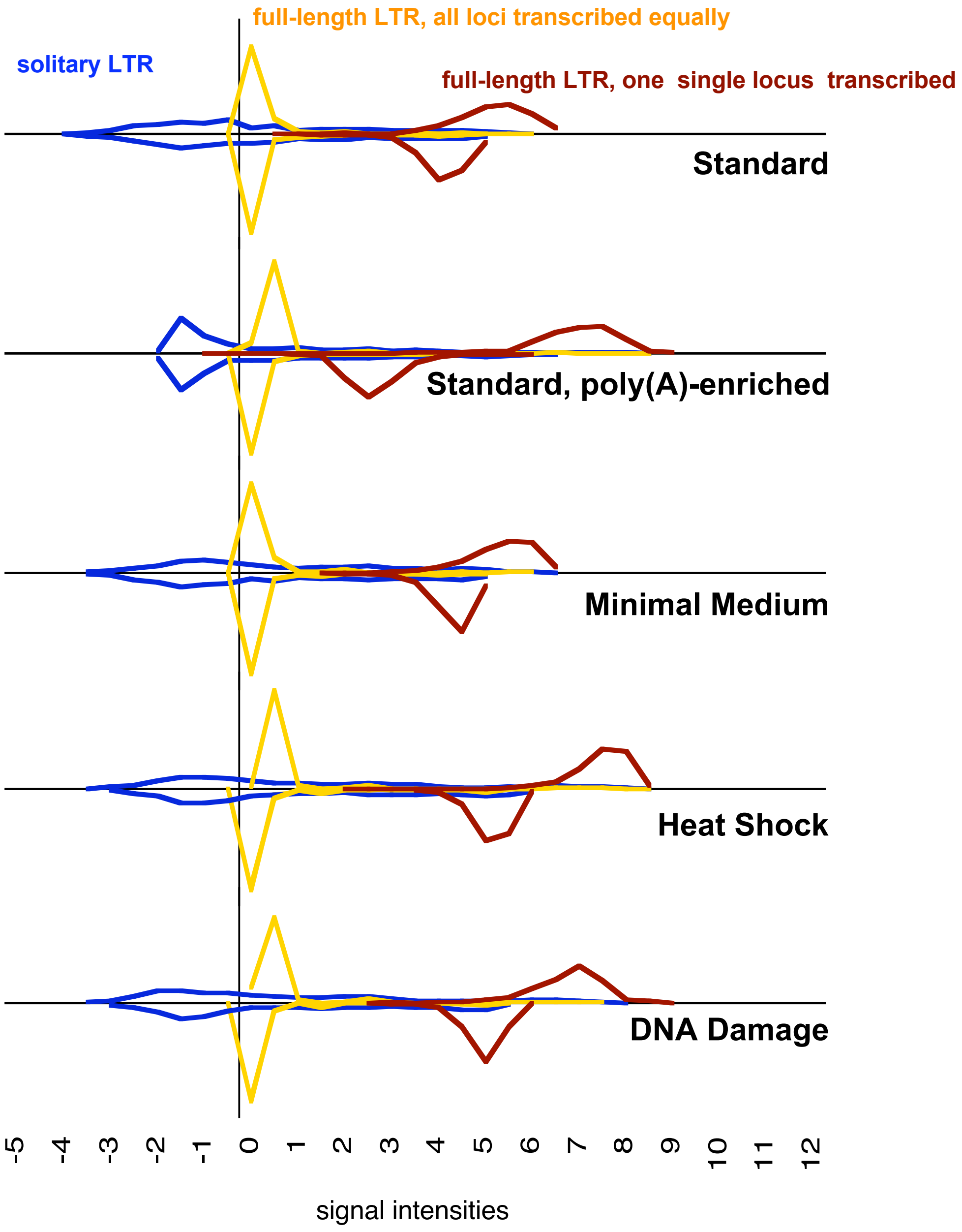
full-length LTR, all loci transcribed equally
solitary LTR
full-length LTR, one single locus transcribed
Standard
Standard, poly(A)-enriched
Minimal Medium
Heat Shock
DNA Damage
-5 -4 -3 -2 -1 0 1 2 3 4 5 6 7 8 9 10 11 12
signal intensities

Figure S6

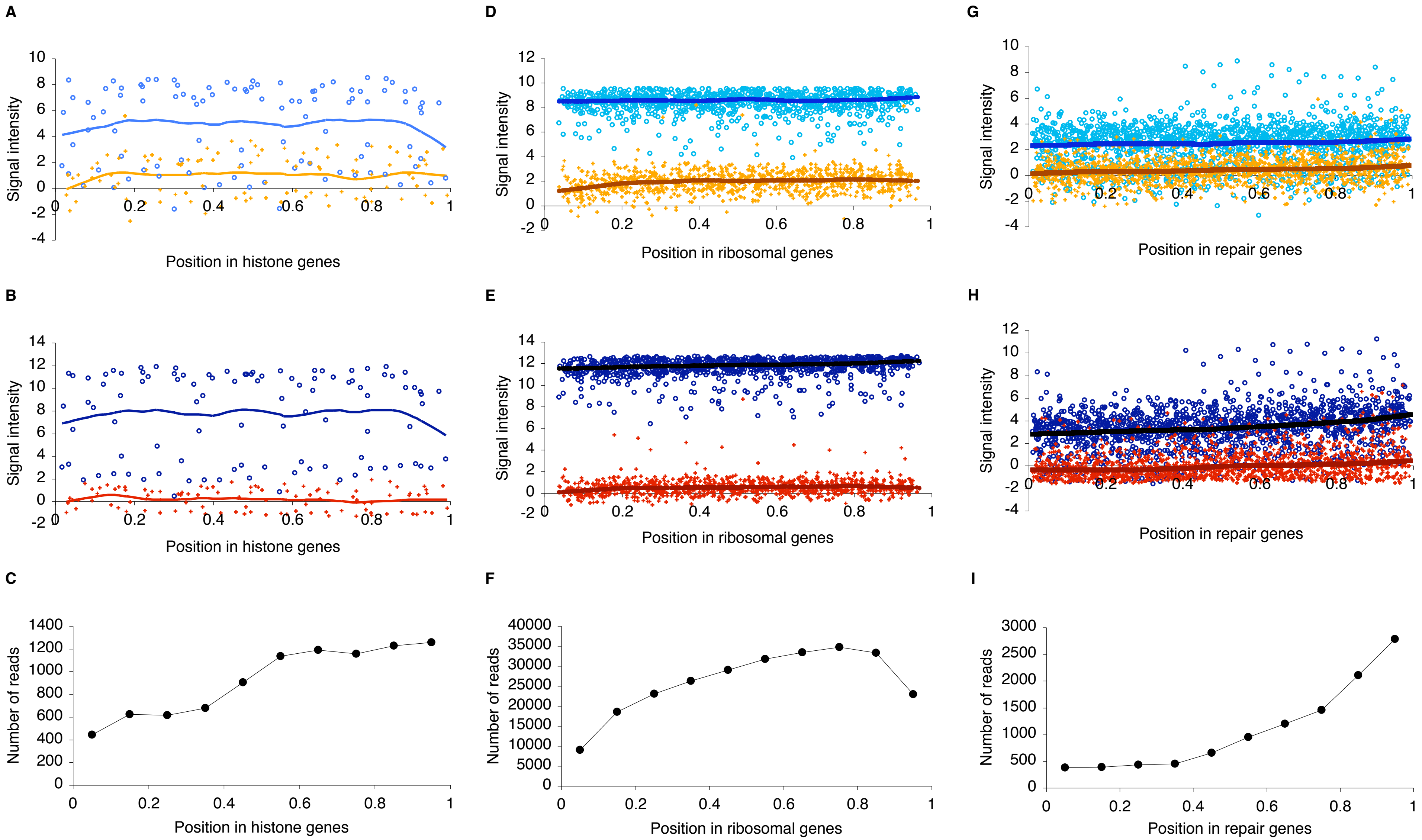

A
Signal intensity
Position in histone genes
B
Signal intensity
Position in histone genes
C
Number of reads
Position in histone genes
D
Signal intensity
Position in ribosomal genes
E
Signal intensity
Position in ribosomal genes
F
Number of reads
Position in ribosomal genes
G
Signal intensity
Position in repair genes
H
Signal intensity
Position in repair genes
I
Number of reads
Position in repair genes

Figure S7

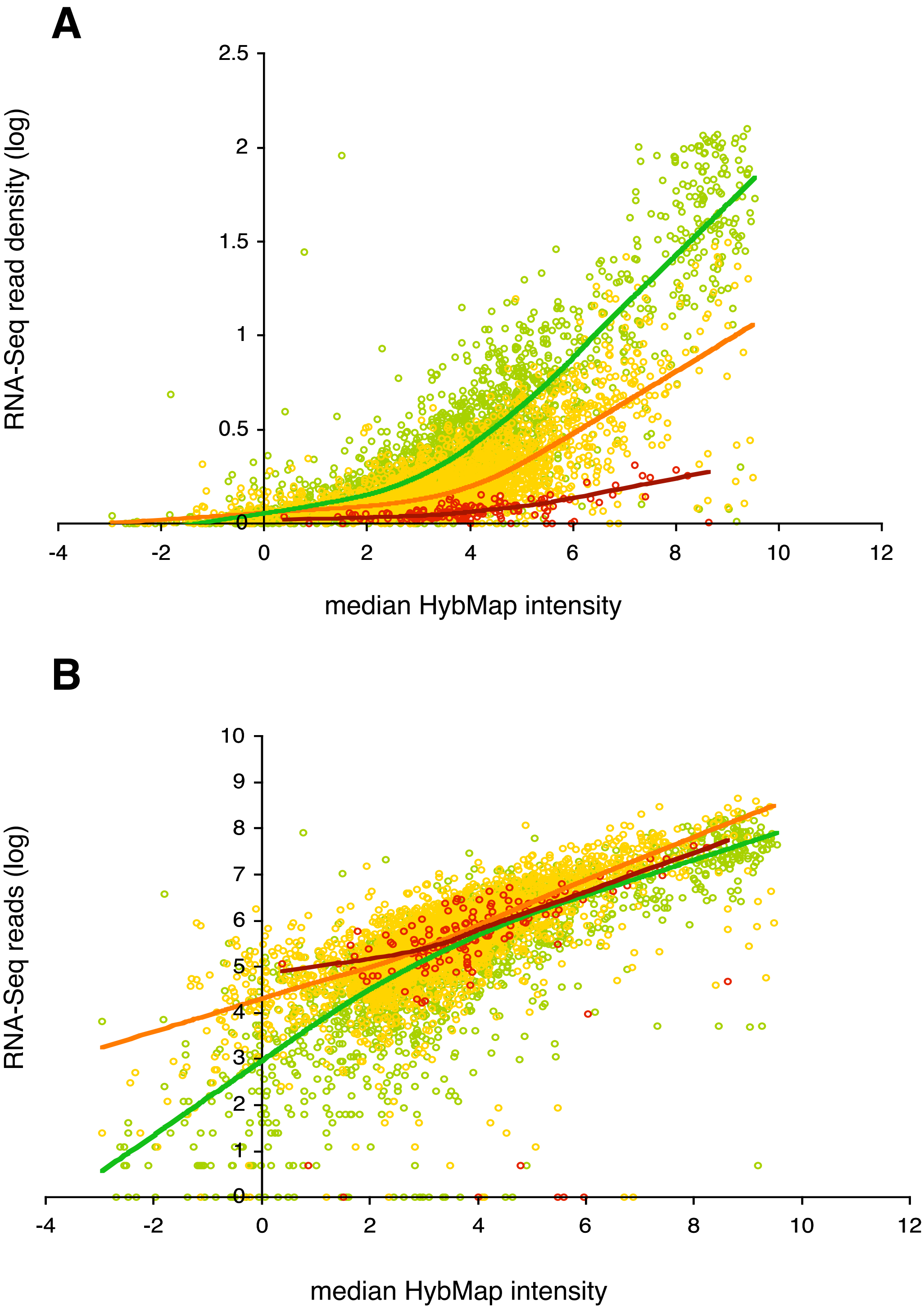
A
RNA-Seq read density (log)
2.5
2
1.5
1
0.5
0
-4
-2
0
2
4
6
8
10
12
median HybMap intensity
B
RNA-Seq reads (log)
10
9
8
7
6
5
4
3
2
1
0
-4
-2
0
2
4
6
8
10
12
median HybMap intensity

Figure S8

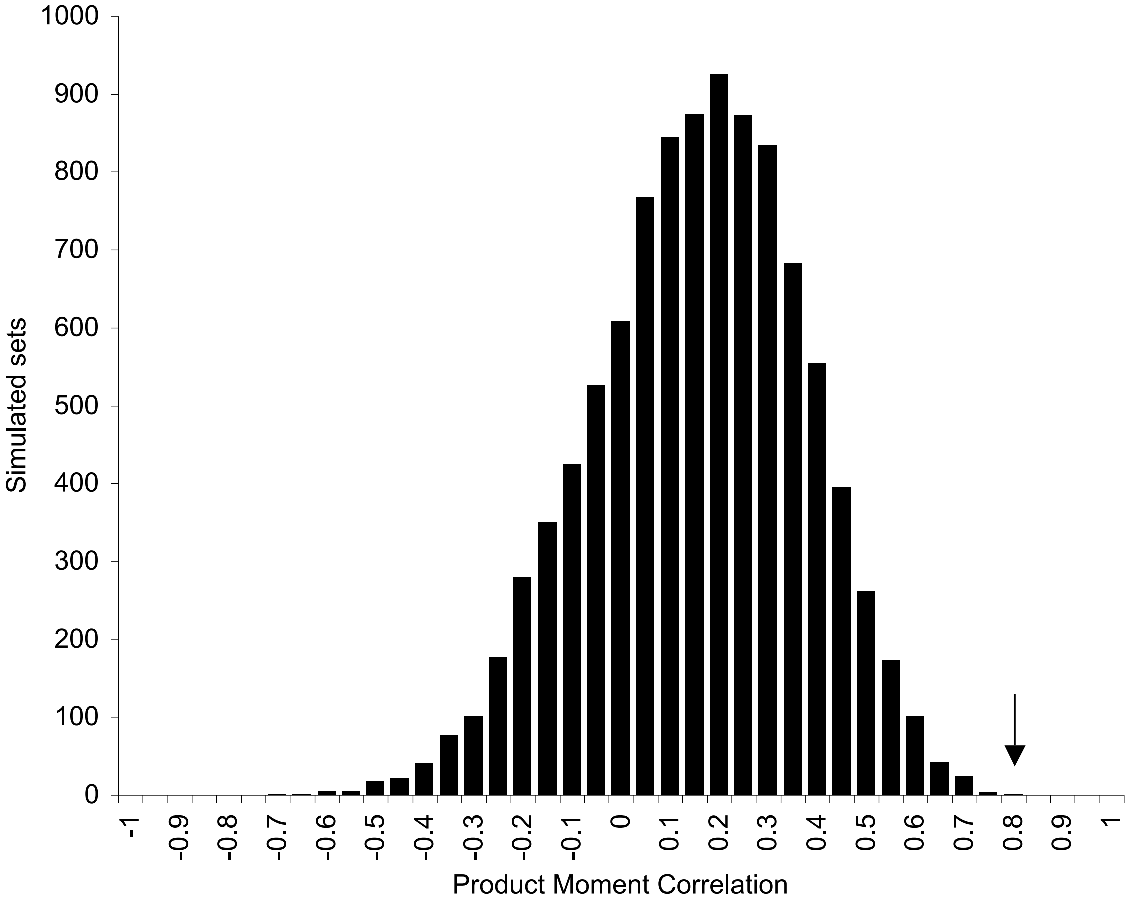

1000
900
800
700
600
500
400
300
200
100
0
Simulated sets
-1
-0.9
-0.8
-0.7
-0.6
-0.5
-0.4
-0.3
-0.2
-0.1
0
0.1
0.2
0.3
0.4
0.5
0.6
0.7
0.8
0.9
1
Product Moment Correlation

Figure S9

A

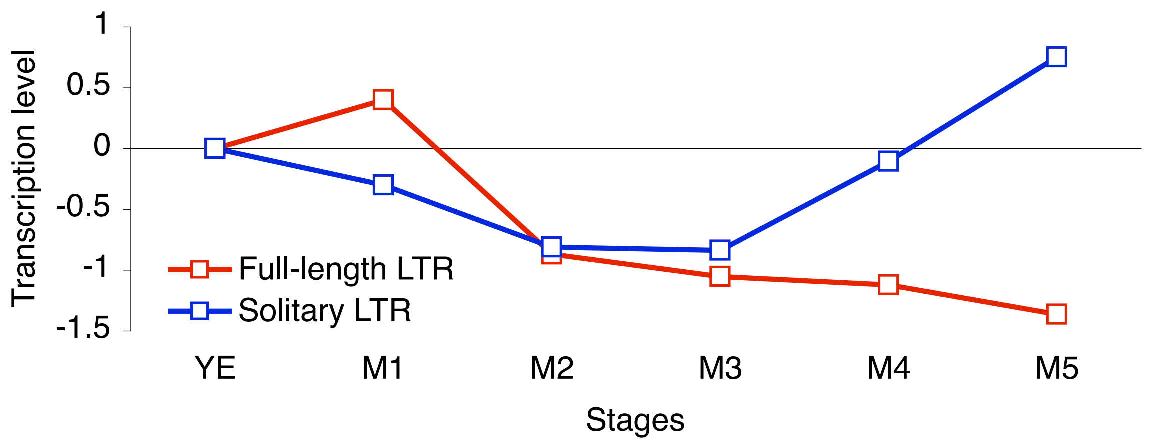

B

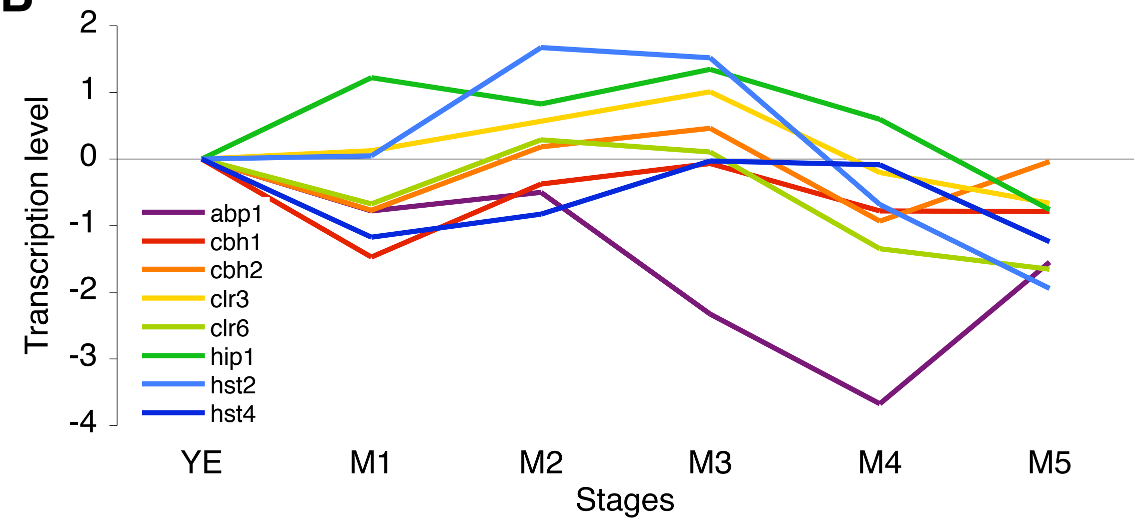

C

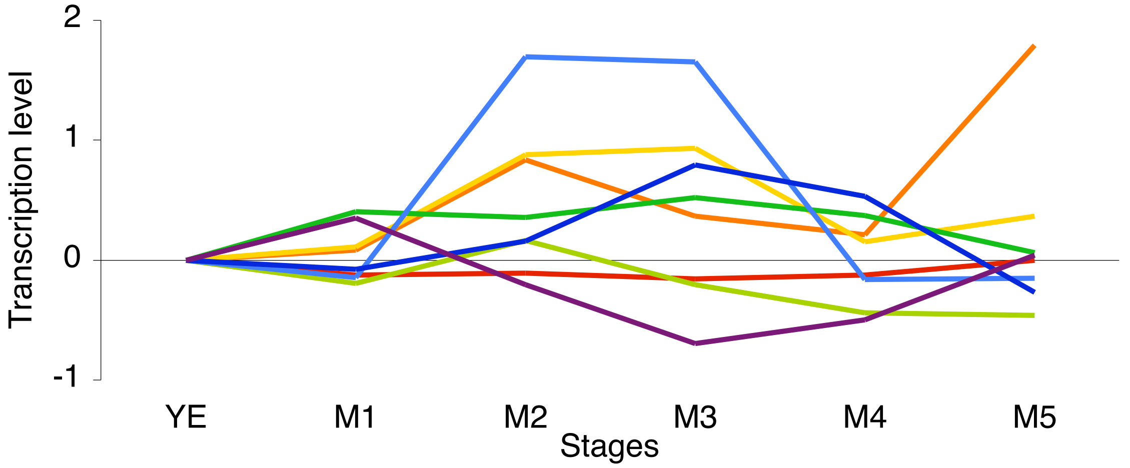

Figure S10

```
LTR_14   TGTCAGCAATACTACATTACGCTATGATACACTACGTTGCGTATCACTATATGT-CACATGTTCTAATTATATATCGTACCA-----TTTATGATACGATATGGAAATTAATCTTAATG-ATA---ATCTATTAAGATCTATATTATCTGAATACTATAAATAG-AGCTACTGCTGAACTC-GTT
LTR_39   ................C.......................................................................G................G..G.............................A.......................................CTC...
LTR_40   .A...........T.A.......A....-T.........................C.......A........T.......G............T..G.C.G.........................T..T.....C...............................A...CTC...
LTR_34   --------..T..TATG.CA..A..AC............A...............................................G...A............G..G.............................A.......................................CTC...
LTR_36   -..T......G...-G..GT.T...A.....T.G.....A.C.....C.....-........C.....C..TC..............G............T..................C.....C.......A......G............C.........-.G............CTC...
LTR_43   ................C......................A................................................G................G..G.............................A.......................................CTC...
LTR_47   ------..........C........A.............A................................................G................G..G.............................A.......................................CTC...
LTR_80   ................C........A.............A................................................G................G..G.............................A.......................................CTC...
LTR_81   ................C.......................................................T.......G..........A..T..G..G.......................................................................CTC...
LTR_83   ................C........A.........A....................................T.......G............T..G..G.......................................................................CTC...
LTR_88   ........G.......C........................................................................G................G..G.......................................................................CTC...
LTR_127  ................C...................-...................................................G................G..G.......................................................................CTC...
LTR_128  ................C........................................................................G................G..G.............................A.......................................CTC...
LTR_129  -----ATT.C.T.GGTG.CA..A..AC..............................................................G................G..G.............................A.......................................CTC...
LTR_134  ................C.......C................................................................G................G..G...............................................A.....................CTC...
LTR_136  ................C........A.............A................................................G................G..G.............................A.......................................CTC...
LTR_140  ...T..AC......T.G..............G.....A.A.A..........A....TG...C........TC..............CG............T.....G..T............G..........C......C..T.A........T........T....A.A....TC...
LTR_144  ................C........A.........A.........................................TT.......G............T..G..G.........A.............C.........................T..................CTC...
LTR_153  ...T...G.......C..T............T..........................................A..T.......G............T..G..G.....................................A.............T................T.......CTC...
LTR_164  ................C......................A................................................G................G..G.............................A.......................................CTC...
LTR_168  ................C......................T................................................G................G..........................................................................CTC...
LTR_173  ................C...........T...........................................................G................G...............................A......................................TCTC...
LTR_175  ................C....T...A....CA.......A.G..........A...........C.T...C......C...G.....CAG.........G.T..T......T....CA................A...C.A..C....A..C.AC..........T.......A...CTCA..
LTR_177  ................C......................A................................................G................G..G.............................A.......................................CTC...
LTR_185  ................C........A...............................................................G................G..G.......................................................................CTC...
LTR_195  ................C........A..............................................A............GC...........T..G..G.......................................................................CTC...
LTR_196  ----------------------------------------.................................................G............T..G..G...............................................A.....................CTC...
LTR_199  ................C........A..............................................A............G............T..G..G.....................C......................A.....................CTC...
LTR_200  ...T.....C......CC.........G.....AG.......................GC............................G............T..G..G.....................C......................A.....................CTC...
LTR_202  -----...........C........A...............................-------......A............G............T..G..G.....................C......................A.....................CTC...
LTR_206  ................C........A.............A................................................G................G..G.............................A.......................................CTC...
LTR_215  ACAACA...C.A..T.C..T.....AC.GT.T.G...CA.ACG..........T.........C.T...C......C...G.....CAG.........G.T..T......T....CA................A...C.A..C....A..C.ACG.........T.......A...CTCA..
LTR_220  ................C..A.........T.T..........T.............................................G............T..T...G........T..A............T..G..........................................CTC...
LTR_221  -----------.G.G....-.T.........G.......A..G...........T.........C.T...C......C...G.....CAG.........G.T..T......T....CA................A...C.A..C....A..C.ACG.........T.......A...CTCA..
LTR_223  ................C......................T................................................G................G..G.............................A.......................................CTC...
LTR_224  ------..........C......................T................................................G................G..G.............................A......................................TCTC...
LTR_227  ..........TG...C.........................................................................G................G..G.............................A.......................................CTC...
LTR_23   ..AATATT..G.CTT.G..T.T.-..T..A.TA.T..CA-.C.AT...GC.CTACG.T...A--C..C..CG.TG....T...C.....A....TC..-..G.TC..CA.C..A.C.TA........-..CG..G.T.A....T.AG---A..G.T.G.........................CTC...
LTR_42   -------.T.......C...AT.....T..---.......T........G...A.......C..A............A............AA...G....T....T..GGC.G.......G..............--...T.........................GG..--AC.TA.A....A.GA.AA
LTR_109  ................C........................................T..............................G................G.........G..........................................................CA.......GA.GA
LTR_123  ................C...................-...................................................G............T..G..G.....................G......................A.......................GA.GA
LTR_125  ................C...................-...................................................G............T..G..G...........G..........................................A.......................GA.GA
LTR_179  -----------------------..................................................................G................G.........................C............................................CA.......GA.GA
LTR_230  ---------------------------....T......C.A...G......................C..G.-C...............AG...............T......G............G...T..G..A.....TCT.GC...TA...........AGAT.......A.......GA.GA
LTR_41   --------------------..A..AC.........A.A...............T.....GG...AC..AT.AAG.T.CGTCTA..G..C..CACGGG.AACT..CCG...AT..AC...GAT.C.CG..G.T.G.A.CTA.T.AGA....T...........T.TTAA.......GA.GA
LTR_154  .A.........TAG..C....T....G....T..T-.......C......G..A....T.....AA.T.AT..GT.ATA.T.CGTTTA..G..A...C.ATGG.AAC.G.CCT...AT..AC...GAT..TC...G-C.AAA.CTA.T.GAA..G.T.........G........TAA.......AA.GA
LTR_181  ................C........................................T.....GG...AC..GC.AAG.T.CGTTTA..G..A..CACGGG.AACT..CCG...AT..ACG..GAT.C.CG..G.T.A.A.CTA.T.AGA....T..........C.T...AA.......GA.GA
LTR_204  ................C........................................T.....GG...AC..GC.AAG.T.CGTTTA..G..A..CACGGG.AACT...CG...AT..AC...GAT.C.CG..G.T.A.A.CTA.T.AGA....T.............T...AA.......GA.GA
LTR_225  ................C....T....C....T...........................T.....GG...AC..GC.AAG.T.CGTTTA..G..A..CACGGG.AACT..CCG...AT..AC...GAT.C.CG..G.T.A.A.CTA.T.AGA....T..........C.T...AA.......GA.GA
LTR_235  .A...A.G..T--...C.....C..A..GT.....A..A..CC....T...........T...C.GAC..ATCT.T.AAG.TTCGTTTA..G..A..TA.GGG.AAGT..CCT...AC...C..GGATTC.CG.G..T.G.A.CTA.T.AGA..C.A-.A...........TTA.........GA.GA
LTR_18   .......................................T.................T.....GG...AC..GC.AAG.T.CGTTTA..G..A..CACGGG.AACT..CC....AT..AC-..GTT.C.AG..G.T.A.A.CTA.T.AGA....T..........C.T...AA......CTC...
LTR_20   ................C........................................T.....GG...AC..GC.AAG.T.CGTTTA..G..A..CACGGG.AACT..CCG...AT..AC...GAT.C.CG..G.T.A.A.CTA.T.AGA....T..........C.T...AA......CTC...
LTR_22   ...T............C........AG....T........A..C..............T.....GG...AC..GC.AAG.T.TATTTA..G..C..CACGGG.AACT..CCG.T.AT...T...AAT.ATCT..G.T.G.A.CTAGTCGGA....T...G........T.C.AA......CTC...
LTR_25   ..........G....C.........................................T.....GG...AC..GC.AAG.T.CGTTTA..G..A..CACGGG.AACT..CCG...AT..AC...GAT.C.CG..G.T.A.A.CTA.T.AGA....T..........C.T...AA......CTC...
LTR_38   ......T.........C........................................T.....GG...AC..GC.AAG.T.CGTTTA..G..A..CACGGG.AACT..CCG...AT..AC...GAT.C.CG..G.T.A.A.CTA.T.AGA....T..........C.T...AA......CTC...
LTR_48   ............C...C........................................T.....GG...AC..GC.AAG.T.CGTTTA..G..A..CACGGG.AACT..CCG...AT..AC...GAT.C.C...G.T.A.A.CTA.T.AGA....T..........C.T...AA......CTC...
LTR_51   ................C........................................T.....GG...AC..GC.AAG.T.CGTTTA..G..A..CACGGG.AACT..CCG...AT..AC...GTT.C.CG..G.T.A.A.CTA.T.AGA....T........C.C.T...AA......CTC...
LTR_53   ................C...................T....................T.....GG...AC..GC.AAG.T.CGTTTA..G..A..CACGGG.AACT..CCG...AT..AC...GAT.C.CG..G.T.A.A.CTA.T.AGA....T..........C.T...AA......CTC...
LTR_66   ..........T....C...................C................C....T.....GG...AC..GC.AAG.T.CGTTTA..G..A..CACGGG.AACT..CCG...AT..AC...GAT.C.CG..G.T.A.A.CTA.T.AGA....T..........C.T...AA.....TCTC...
LTR_76   ................C........................................T.....GG...AC..GC.AAG.T.CGTTTA..G..A..CACGGG.AACT..CCG...AT..AC...GAT.C.CG..G.T.A.A.CTA.T.AGA....T..........C.T...AA......CTC...
LTR_107  ..C..TT....TA..GC..T......................................T.....GG...AC..GC.AAG.T.CGTTTA..G..A..CACGGG.AATT..CCG...AT..AC...GAT.C.CG....T.A.A.CTA.T.AGA....T..........C.T...AA......CTC...
LTR_108  ................C........................................T.....GG...AC..GC.AAG.T.CGTTTA..G..A..CACGGG.AACT..CCG...AT..AC...GAT.C.CG....T.A.A.CTA.T.AGA....T..........C.T...AA......CTC...
LTR_126  ..........T.....C........A...T.G..........................T.....GG...AC..GC.AAG.T.CGTTTA..G..A..CACGGG.AACT..CTG...AT.CAC---.AT.CTCGC.G.T.A.A.CTA.T.AGA....T............T.C.AA......CTC...
LTR_131  ................C.........C...............................T.....GG...AC..GC.AAG.T.CGTTTA..G..A..CACGGG.AACT..CCT...AT..AC...GAT.C.CG..G.T.A.A.CTA.T.AGA....T..........C.T...AA......CTC...
LTR_148  ................C...........T..................GA....T.....GG...AC..GC.AAG.T.CGTTTA..G..A..CATGGG.AACT..CCG...AT..AC...GAT.C.CG..G.T.A.A.CTA.T.AGA....T..........C.T...GA.....TCTC...
LTR_157  --------------TG.C.-..A..AC.............C................T.....GG...AC..GC.AAG.T.CGTTTA..G..A..CACGGGAAACT..CCG...AT..AA...AAT.C.CG..G.T.A.A.CTA.T.AGA....T..........C.T...AA......CTC...
LTR_169  ......G....G....C.........C...............................T.....GG...AC..GC.AAG.T.CGTTTA..G..A..CACGGG.AACT..CCG...AT..AC...GAT.C.CG..G.T.A.A.CTA.T.AGA....T..........C.T...AA......CTC...
LTR_180  -----------------------......G.A....A.T..................T.....GA...AC..GT.AAG.T.CGCTTG..G..A..CAC.GG.AACT...CG...ATG.AC...AAT.C.CG..G.T.A.AGCCA.T.A---------........C.T...AA......CTC...
LTR_193  ........A.G....C.........T....G..T....................T..A.-GG...AC..GTCAAG.T.CGCTTA..G..C..CACGGG.AACT..CCG...AT..TC...GAT.C.CG..G.T.A.A...TACT.AGA....T............T.C.AA......CTC...
LTR_210  ................C..........................C..............T.....GG...AC..GC.AAG.T.CGTTTA..G..A..CACGGG.AACT..CCG...AT..AC...GAT.C.CGA.G.T.A.A.CTA.T.AGA....T...G........T.C.AA......CTC...
LTR_217  ................C.........C...............................T.....GG...AC..GC.AAG.T.CGTTTA..G..A..CACGGG.AACT..CCT...AT..AC...GAT.C.CG..G.T.-.A.GTA.T.AGA....T..........C.T...AA......CTC...
```

Figure S10

```
LTR_14   CCTCAGTTCAGTTATGAGCTATATTAGTGATAGGTAACAT--TATAACCCAGTTAATACAATACCTATACTCAGTTGCTACTTATACAACCTGTGTATTGTAATATAA-TAGATCA-CAAGGAAAACTCACCGCAGTTCTACGTA-TCCTTAAATCAGATACCAAACTGCGTAGCTTACA
LTR_39   ..............................................................................................................C.....................................................G............T.....AA...
LTR_40   ..............CA.......CC.........................TT...............C..................TT.T...........CG..-TTA...G..................................A.....A.GA....G.............A...
LTR_34   .................................T....................................................................................................................................................
LTR_36   ...............A....G.............................T..A..G..T.......G........C..........TT.....C..T.C......-T.A...G..........................-T.........G.....G......T..............G...
LTR_43   ..........................................................................................................................................................T........................
LTR_47   ....................................................................................................................................................................................
LTR_80   ....................................................................................................................................................................................
LTR_81   .................................................................................................................................T.A................................A..........
LTR_88   ......................................................................................T..............................................A...............................................
LTR_127  ..........................................C......T..................TC.............................................................T.......C...........................................
LTR_128  ....................................................................................................................................................................................
LTR_129  ....................................................................................................................................................................................
LTR_134  ..............................................................................................................T.....................................................................
LTR_136  ....................................................................................................................................................................................
LTR_140  .G.............A......G..........-....GC.......GTTG.A..G..T....T.......T..............TT....T.A...C.C...T-T.A......G......A...C..........A...AG.......G.....TATG....TT.............G...
LTR_144  ...............AG.......C.........................T.T.....................................TT............CG..-T.A....CG......................T...A.....G.......A.GA....G.............C...
LTR_153  ...............A..................................T..............................................G....................................A................A........T.................A...
LTR_164  ....................................................................................................................................................T...............................
LTR_168  ...............................................................................................C....................................................................................
LTR_173  .................................................................................................................................TG......T......G.C...AT...........G.........G...
LTR_175  ...T.-CA..A....A..T.G....T..A...AA.....A....C..TTT-.A..G...........A...................T.....................---.--AG.T.A.........A.T............T..TA..G.....GT........................
LTR_177  ....................................................................................................................G...............................................................
LTR_185  ....................................................................................................................................................................................
LTR_195  .................................................T.A..............................C...T..........A........G..............................A................................T.........G...
LTR_196  ................................................T..........................................................................................................................T.............
LTR_199  ...............A................................T...................................................................................................................................
LTR_200  ...............A..................................T.A.............................C...T.............C....-TGA.....TG..G......-..T.................CTT.....A..T...TA.................G...
LTR_202  .................................................T.A..............................C...T..........A.............G..............................................................A...
LTR_206  ....................................................................................................................................................................................
LTR_215  ...T.-CA..A....A..T.G....T.CA...AA.....A....C...TT-.A..G...........C..................TC......C.......C....AT.A...GA.G.A.T.AG.......T....A...A.T.....TG......G...........................G.T.
LTR_220  ....................................................................................................................................................T........................CG...
LTR_221  ...T.-CA..A....A..T.G....T.CA.....A.....A....C...TT-.A..G...........A..................GT....................-TCA...G...CA........A.T............T...A..G......G...........T.........G...
LTR_223  ....................................................................................................................................................................................
LTR_224  .................................................................................................................................T..C.............................T..........
LTR_227  .................................................................................................................................T.........................................G....
LTR_23   T..G......A.......................................................................C.A.........T..............A...................G................................................G...
LTR_42   A.GAG..........A.AT..C............A.....AA........T.CA.....G.........-......A......AA...TT........G...C....--TAGTAG....G............A.......T.G.A.....G.....-T.....T..............G...
LTR_109  A.GAG...................................................................................................A....C..............................................................---
LTR_123  A.GAG.............................................................................C...T.............................................................................................
LTR_125  A.GAG...............................................................T...............................................................................................................
LTR_179  A.GAG..................C............................................................................................................................................................
LTR_230  AAGAG.......CA..A.....C...T.......A.....A.......C..TTTA..T..T...........TGA...T.....T..T.....A......AC....-TTA...G..G.........G.........T.A..........G.....AT.AT...A...T...A...TAA...
LTR_41   A.GAG..........A........................TT.......T................................C....G.......................C.......................................T.......A.......T......AG..
LTR_154  A.GAG..........A..T...G.A...........G..G......CTAT.CA.....G.............G.............TT.......CA....TA..........AG.................TTA.....G.........A.....A.......TTGGT...TA...T.A...
LTR_181  A.GAG...................................T.-..................................................C....................................................................................
LTR_204  A.GAG...................................T.-....T...............................................................C......................C...........................................
LTR_225  A.GAG...................................T.-......T......................T.......................................C..................................................................
LTR_235  A.GA....T......A........................A...C.G.AT.TA.GG..T.....-...TG.AC..A..GT......TT.....CA.....C..........TG..................T......T..........G....A...................A.G...
LTR_18   ........................................T.-.....A...............------............C...........T.................G.T.....................-.........................................
LTR_20   ........................................T.-...G................................................................C..........................T.......................................
LTR_22   ...............A........................T.-....T.T.................G.................TT.......T.G...C..........AG.............-.................................A....A.............
LTR_25   ........................................T.-.....T..............................................................C..................................................................
LTR_38   ........................................T.-....................................................................C.........................................G........................
LTR_48   ........................................T.-....................................................................C..........................................A.......................
LTR_51   ........................................T.-....................................................................C..................................................................
LTR_53   ........................................T.-....................................................................C.......................C............................A.............
LTR_66   ........................................T.-.........................................................................................................................................
LTR_76   ........................................T.-....T...............................................................C..................................................................
LTR_107  ........................................T.-.............................................................A....C...T......................C................................A.-----
LTR_108  ........................................T.-.............................................................A....C...T..............................................................
LTR_126  ........T......A........C...............C.-......T...................TC.............................................................C................-..A..................G.T.
LTR_131  ........................................T.-....................................................................C..................................................................
LTR_148  ........................................T.-....................................................................C.................TA....................T........................
LTR_157  .........G..............................T.-.......................................C..-.......................................T.............G.........-..........................
LTR_169  ........................................T.-....................................................................C..................................................................
LTR_180  .......................C................T.-.....................................................G...C.................................A..T...A.T...G.....GTG.......G.-..GA...T.A..-
LTR_193  .............................G..........T.-......T....................................TC..A.........C..........................................................................A.A.
LTR_210  ........................................T.-.......T.................T............................A...............C........................................C.........................
LTR_217  ........................................T.-....................................................................C..................................................................
```
